# Association of Selected STAT Inhibitors with Prolactin-Induced Protein (PIP) in Breast Cancer

**DOI:** 10.3390/ijms26041416

**Published:** 2025-02-07

**Authors:** Karolina Jabłońska, Alicja Kmiecik, Katarzyna Nowińska, Aleksandra Piotrowska, Jarosław Suchański, Katarzyna Ratajczak-Wielgomas, Aleksandra Partyńska, Hanna Romanowicz, Beata Smolarz, Rafał Matkowski, Piotr Dzięgiel

**Affiliations:** 1 Division of Histology and Embryology, Department of Human Morphology and Embryology, Faculty of Medicine, Wroclaw Medical University, 50-368 Wroclaw, Poland; alicja.kmiecik@umw.edu.pl (A.K.); katarzyna.nowinska@umw.edu.pl (K.N.); aleksandra.piotrowska@umw.edu.pl (A.P.); katarzyna.ratajczak-wielgomas@umw.edu.pl (K.R.-W.); aleksandra.partynska@umw.edu.pl (A.P.); piotr.dziegiel@umw.edu.pl (P.D.); 2 Department of Biochemistry and Molecular Biology, Wroclaw University of Environmental and Life Sciences, 50-375 Wroclaw, Poland; jaroslaw.suchanski@upwr.edu.pl; 3 Department of Pathology, Polish Mother Memorial Hospital-Research Institute, 93-338 Lodz, Poland; hanna-romanowicz@wp.pl (H.R.); smolbea@wp.pl (B.S.); 4 Department of Oncology, Faculty of Medicine, Wroclaw Medical University, 50-367 Wroclaw, Poland; rafal.matkowski@umw.edu.pl; 5 Lower Silesian Oncology, Pulmonology and Hematology Center, 53-413 Wroclaw, Poland; 6 Department of Human Biology, Faculty of Physiotherapy, Wroclaw University of Health and Sport Sciences, 51-612 Wroclaw, Poland

**Keywords:** breast cancer, prolactin-induced protein, PIP, GCDFP-15, signal transducer and activator of transcription, STAT, PIAS3, SOCS3, prolactin

## Abstract

Breast cancer (BC) is the most common cancer in women, and a higher level of prolactin-induced protein (PIP) is associated with better responses to adjuvant chemotherapy. The signal transducer and activator of transcription 5 (STAT5) is a potential regulator of the PIP gene. Prolactin (PRL) and its receptor (PRLR) activate JAK2/STAT5 signaling in BC, which is modulated by inhibitors like suppressors of cytokine signaling (SOCS) proteins and protein inhibitors of activated STAT (PIAS). Using real-time PCR and immunohistochemistry, we studied the relationship between PIP and STAT5 inhibitors in BC. Our findings indicated that PIP and STAT5 levels decrease with a higher tumor grade, size, and tumor/nodes/metastasis (TNM) clinical stage, while nuclear PIAS3 levels increase with tumor progression. Both STAT inhibitors are linked to estrogen and progesterone receptor status. Notably, STAT5 correlates positively with PIP, SOCS3, and PIAS3, suggesting that it may be a favorable prognostic factor. Among the STAT inhibitors, only nuclear PIAS3 expression correlates with PIP. In vitro studies indicated that silencing PIAS3 in T47D cells does not affect PIP expression or sensitivity to doxorubicin (DOX), but T47D control cells with a higher PIP expression are more sensitive to DOX, highlighting the need for further investigation into these mechanisms.

## 1. Introduction

Breast cancer (BC) is the most prevalent cancer among women and the second leading cause of cancer-related deaths in women [1]. As reported by the International Agency for Cancer Research, in 2022, there were about 2.31 million newly diagnosed female BC cases worldwide [1]. Although hereditary and genetic factors account for 5–10% of BC cases, studies have shown that nonhereditary factors are the major drivers of this type of cancer. Despite well-developed diagnostics and modern treatment regimens, most patients with advanced BC develop treatment resistance [2].

In our published study, we showed that a high level of prolactin-induced protein (PIP) was positively correlated with the response of BC patients to standard adjuvant chemotherapy [3]. PIP (GCDFP-15, gp-17, EP-GP) was first described as gross cystic disease fluid protein 15 present in the cystic fluid from mastopathy and as a glycoprotein in the culture medium from BC T47D cells [4,5]. PIP is a secretory protein present in seminal plasma, saliva, lacrimal fluid, tears, sweat gland secretion, amniotic fluid, and the blood of pregnant women [6]. The PIP gene is located on chromosome 7.q34 and includes 7000 bp. Its theoretical molecular mass is 13,506 kDa [4]. The PIP gene expression on the levels of mRNA and protein is increased by androgens, progesterone, glucocorticosteroids, prolactin (PRL), growth hormone (GH), and cytokines such as interleukin (IL)-1α, IL-4, and IL-13 and decreased by 17β-estradiol and IL-6 [7,8,9,10].

PIP overexpression has been shown in primary and metastatic BCs, as well as in some breast carcinoma cell lines [3,11,12]. PIP expression has been suggested as a marker for BC and is utilized to identify breast origin in metastatic carcinomas of unknown primary origins [13]. DNA microarray analysis revealed that the expression of the PIP gene was significantly lower in cases of invasive ductal carcinoma (IDC), which poorly responded to standard adjuvant chemotherapy [3]. This allowed us to hypothesize that PIP expression may impact the sensitivity of BC cells to cytostatic agents [14]. Our recent study showed that the increased sensitivity of BC cells to anticancer drugs is due to the proapoptotic activity of PIP [14]. However, the exact functions and regulatory mechanisms of PIP expression in mammary tumor progression remain unclear [3,15].

There is evidence that PIP is primarily regulated at the transcriptional level by the action of PRL and 5α-dihydrotestosterone (DHT), which bind, respectively, to the prolactin (PRLR) and androgen (AR) receptors [16]. PRL binding to the long isoform of PRLR results in receptor dimerization, the activation of JAK2 kinase, and the phosphorylation of tyrosine residues. This promotes the binding and phosphorylation of the signal transducer and activator of transcription (STAT) protein family. STATs, after PRLR dimerization, are translocated from submembranous localization into the nucleus, where they activate genes with promoters containing γ-interferon-sensitive (GAS) elements [17]. Seven mammalian STAT proteins (STAT 1, -2, -3, -4, -5A, -5B, and -6) have been identified. The STAT proteins involved in PRL signaling are STAT5A, STAT5B, STAT1, and STAT3 [18,19].

Debily et al. indicated STAT5 as a potential PIP gene expression transcriptional regulator [20]. STAT5 was first identified as a mammary gland factor (MGF) activated by PRL in the mammary glands of lactating animals [21]. It is now recognized to be activated by many cytokines, including GH, as well as several other ILs and growth factors [22,23]. STAT5A and STAT5B are two highly related proteins with a 96% sequence homology encoded by two separate but clustered genes. These proteins differ in their carboxyl-terminal region, which is involved in transactivation and DNA-binding activities [18,19]. Peck et al. showed that STAT5A, but not STAT5B, is often lost during BC development [24].

STAT signaling is regulated by endogenous inhibitors, namely suppressors of cytokine signaling (SOCS) proteins, phosphatases, and protein inhibitors of activated STAT (PIAS) proteins. In tumorigenesis, STAT proteins are constitutively active and promote the expression of pro-growth and pro-survival genes. Several studies have illustrated that blocking STAT3 or 5 signaling leads to tumor cell apoptosis [25]. Although STAT activation has been widely implicated in cancer, therapeutic STAT inhibitors are still largely absent in the clinic. SOCS1 and SOCS3 terminate signaling from both long and short PRLR forms. In contrast, the PIAS protein terminates the signaling from the long isoform only by interacting with the DNA-binding domain of STAT5 [17].

The SOCS family includes SOCS1-7. SOCS proteins bind near the STAT5-binding site of PRLR and prevent STAT5 interaction with the PRLR long isoform. Additionally, the SOCS box plays a part in forming the E3 ubiquitin ligase complex. STAT proteins also strictly contribute to the upregulation of SOCS gene expression [17,26]. Helman et al. showed that only SOCS3 participates in the termination of the PRLR cycle [27]. Other authors have demonstrated that SOCS1 and SOCS3 are expressed much earlier than SOCS2 in human T47D cells in response to PRL [28]. The mechanism of the SOCS-mediated inhibition of PRL signaling and its role in regulating PIP expression remains unclear.

The PIAS family includes the PIAS1-4 proteins. PIAS regulates the activity of many transcription factors (e.g., STAT proteins, NFkB, SMADs, and the tumor suppressor p53) through several of the following mechanisms: blocking binding to DNA, recruiting co-repressors or co-activators, and participating in protein sumoylation [10,29,30]. PIAS3 suppresses the STAT5 cascade by interacting with the DNA-binding STAT5 domain, but it is still unknown whether STAT5 is sumoylated under PIAS3′s influence [31,32]. The involvement of PIAS3 has been described in several types of cancer, including breast, ovary, and prostate cancer. PIAS3 suppresses the transcriptional activity of STAT5 in BC cells incubated with PRL [33]. PIAS3 overexpression in BC cell lines can significantly modulate STAT5-mediated gene expression and induce cellular apoptosis. The distribution of PIAS3 noted in breast tissues is related to its associations and resides in the cytoplasm and nucleus. A study examining tumors noted increased PIAS3 expression as a function of malignant transformation [16]. The role of PIAS3 in the regulation of PIP expression is still unclear, highlighting a critical area for further investigation. 

Inhibitors targeting STAT molecules, particularly STAT3 and STAT5, are under intensive study. The recent discovery of JAK/STAT as a pathway of cancer resistance has strengthened the scientific rationale for using STAT inhibitors, as well as the combination of STAT inhibitors with other therapies. This therapeutic strategy is expected to result in a broad range of clinical applications for STAT inhibitors. Due to the discrepancies between the association of the PIP function as pro-cancerous and the mechanism of PIP regulation, we decided to verify the relations between the levels of SOCS3 and PIAS3 inhibitors and the PIP expression in BC cells. Moreover, the relationship between the obtained results and clinicopathological data was examined. The control group for these studies consisted of breasts with mastopathy, which is also known as breast dysplasia. Since mastopathy is non-cancerous, it provides a clear contrast to malignant conditions, aiding in identifying specific markers and characteristics related to BC. Using mastopathy breasts as a control group helps to distinguish between benign and malignant changes, improving diagnostic accuracy and treatment strategies.

## 2. Results

**Figure 1:**
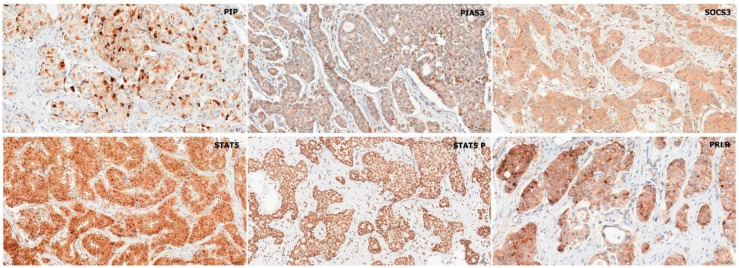
Immunohistochemical analysis of PIP, PIAS3, SOCS3, STAT5, STAT5-P, and PRLR expression. Magnification ×200. PIAS3, protein inhibitor of activated STAT3; PIP, prolactin-induced protein; PRLR, prolactin receptor; SOCS3, suppressor of cytokine signaling 3; STAT5, signal transducer and activator of transcription 5; STAT5-P, phosphorylated signal transducer and activator of transcription 5.

In the analyzed BC cases, antibodies indicated the cytoplasmic localization of PIP and SOCS3. Both cytoplasmic and nuclear expressions were demonstrated for PIAS3, STAT5, and STAT5-P (predominantly nuclear). Membranous localization was noted for PRLR, as shown in Figure 1. Positive immunohistochemical (IHC) expression was noted in over 70% of BC cases for PIAS3 (cytoplasm), SOCS3, PRLR, and STAT5 antigens and in 68% of BC cases for PIP. Among IDC samples, positive nuclear localization in BC cells was noted in the case of PIAS3 (41%), STAT5 (79%), and STAT5-P (92%) antigens. The mean ± standard deviation (SD) values of the immunohistochemical levels of the analyzed antigens were, respectively, PIP (3.13 ± 3.51), PIAS3 (cytoplasmic) (7.75 ± 3.4), PIAS3 (nuclear) (1.47 ± 0.5), SOCS3 (7.28 ± 3.5), STAT5 (cytoplasmic) (6.43 ± 4.43), STAT5 (nuclear) (1.77 ± 0.41), STAT5-P (nuclear) (2.53 ± 1.24), and PRLR (5.99 ± 2.83).

### 2.1. Immunohistochemistry

#### 2.1.1. Association of PIP, PIAS3, SOCS3, STAT5, and PRLR with Clinicopathological Factors 

**Figure 2:**
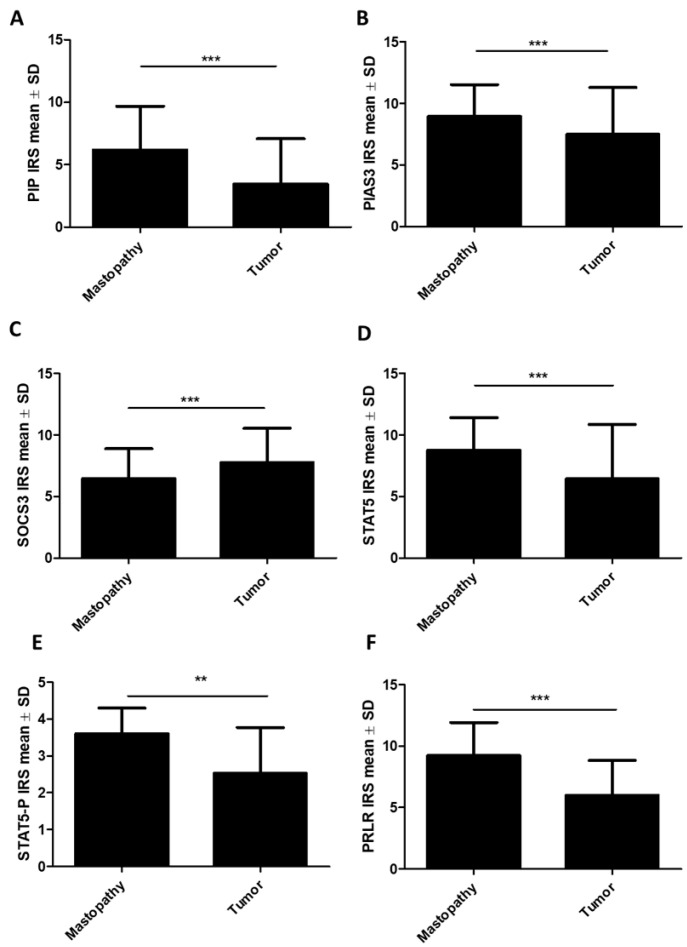
Comparison of immunohistochemical expression of (**A**) PIP, (**B**) PIAS3 (cytoplasmic), (**C**) SOCS3, (**D**) STAT5 (cytoplasmic), (**E**) STAT5-P, and (**F**) PRLR expression in BC tissue (*n* = 554) to the control (mastopathy) (*n* = 61). ** *p* < 0.01, *** *p* < 0.001, Mann–Whitney U test. IRS, immunoreactive score; PIAS3, protein inhibitor of activated STAT3; PIP, prolactin-induced protein; PRLR, prolactin receptor; SD, standard deviation; SOCS3, suppressor of cytokine signaling 3; STAT5, signal transducer and activator of transcription 5; STAT5-P, phosphorylated signal transducer and activator of transcription 5.

Statistical analyses of the associations of PIP, PIAS3, SOCS3, STAT5, and PRLR expression in BC cells with clinical and pathological factors were performed. To compare the mean levels of the tested proteins between tumors and the control group (mastopathy), the Mann–Whitney U test was used. The immunoexpression levels in mastopathy vs. tumor of PIP (6.17 ± 3.52 vs. 3.44 ± 3.52, *p* < 0.001), PIAS3 (cytoplasmic) (8.94 ± 2.59 vs. 7.49 ± 3.81, *p* < 0.001), STAT5 (cytoplasmic) (8.77 ± 2.63 vs. 6.43 ± 4.43, *p* < 0.001), STAT5-P (3.6 ± 0.7 vs. 2.53 ± 1.24, *p* < 0.01), and PRLR (9.22 ± 2.7 vs. 5.99 ± 2.83, *p* < 0.001) (Figure 2A,B,D–F) were significantly lower in tumors (*n* = 554) than in mastopathy (*n* = 61). In the case of SOCS3, there was a significantly higher immunoexpression level in cancer in comparison to the control (mastopathy vs. tumor: 6.46 ± 2.42 vs. 7.77 ± 2.77, *p* < 0.001) (Figure 2C). In the case of PIAS3 (nuclear) and STAT5 (nuclear), we did not observe statistically significant differences.

**Figure 3:**
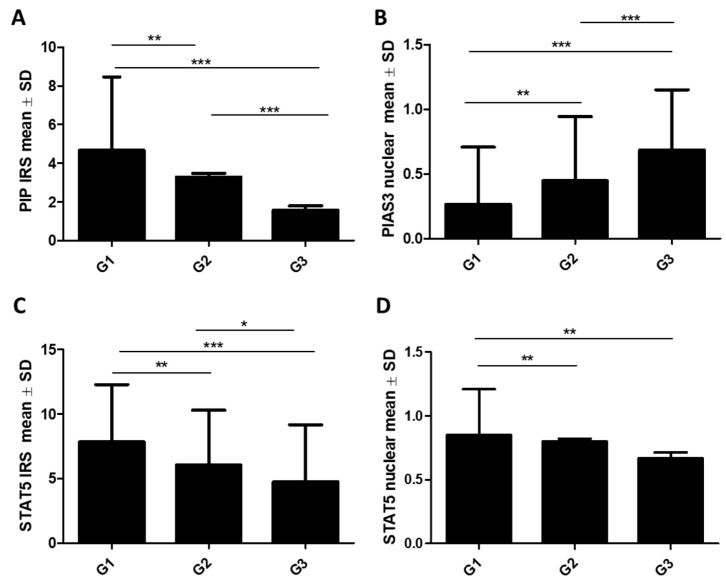
Comparison of (**A**) PIP, (**B**) PIAS3 (nuclear), (**C**) STAT5 (cytoplasmic), and (**D**) STAT5 (nuclear) expression levels detected by immunohistochemistry according to histological grade G (G1 *n* = 89, G2 *n* = 299, G3 *n* = 166). * *p* < 0.05, ** *p* < 0.01, *** *p* < 0.001, Kruskal–Wallis test with Dunn’s multiple comparison test. G, grade of malignancy; IRS, immunoreactive score; PIAS3, protein inhibitor of activated STAT3; PIP, prolactin-induced protein; SD, standard deviation, STAT5, signal transducer and activator of transcription 5.

To compare the mean ± SD of the tested proteins across histological grade G, we used the Kruskal–Wallis test followed by Dunn’s multiple comparison test. Analysis of the obtained results showed a statistically significant decrease in the level of PIP protein expression (G1: 4.64 ± 3.8, G2: 3.29 ± 3.6, G3: 1.58 ± 2.38, *p* < 0.001; G1 vs. G2: *p* < 0.01, G1 vs. G3: *p* < 0.001, G2 vs. G3: *p* < 0.001), STAT5 (cytoplasmic) (G1: 7.85 ± 4.43, G2: 6.04 ± 4.25, G3: 4.74 ± 4.42, *p* < 0.001; G1 vs. G2: *p* < 0.01, G1 vs. G3: *p* < 0.001, G2 vs. G3: *p* < 0.05), and STAT5 (nuclear) (G1: 0.85 ± 0.36, G2: 0.8 ± 0.39, G3: 0.67 ± 0.47, *p* = 0.0024; G1 vs. G2: *p* < 0.01, G1 vs. G3: *p* < 0.01) with the histological grade of the tumors (G) (G1 *n* = 89, G2 *n* = 299, G3 *n* = 166) (Figure 3A,C,D). However, the PIAS3 (nuclear) level significantly increased with tumor histology grade (G1: 0.26 ± 0.44, G2: 0.45 ± 0.49, G3: 0.68 ± 0.46, *p* < 0.001; G1 vs. G2: *p* < 0.01, G1 vs. G3: *p* < 0.001, G2 vs. G3: *p* < 0.001) (Figure 3B). In the case of PIAS3 (cytoplasmic), SOCS3, STAT5-P, and PRLR, there were no significant relationships with G.

**Figure 4:**
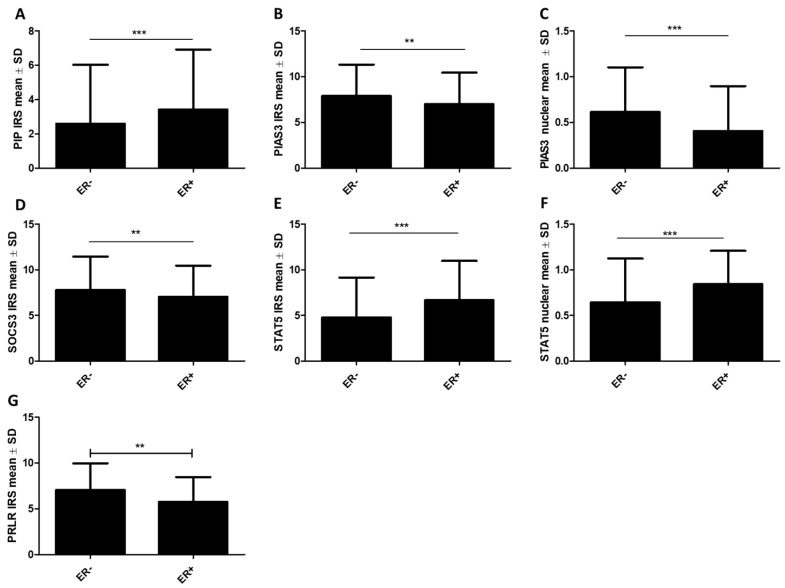
Protein expression levels of (**A**) PIP, (**B**) PIAS3 (cytoplasmic), (**C**) PIAS3 (nuclear), (**D**) SOCS3, (**E**) STAT5 (cytoplasmic), (**F**) STAT5 (nuclear), and (**G**) PRLR according to the status of estrogen receptors (ER) (ER− *n* = 144, ER+ *n* = 410). ** *p* < 0.01, *** *p* < 0.001, Mann–Whitney U test. ER, estrogen receptor; IRS, immunoreactive score; PIAS3, protein inhibitor of activated STAT3; PIP, prolactin-induced protein; PRLR, prolactin receptor; SD, standard deviation; SOCS3, suppressor of cytokine signaling 3; STAT5, signal transducer and activator of transcription 5.

**Figure 5:**
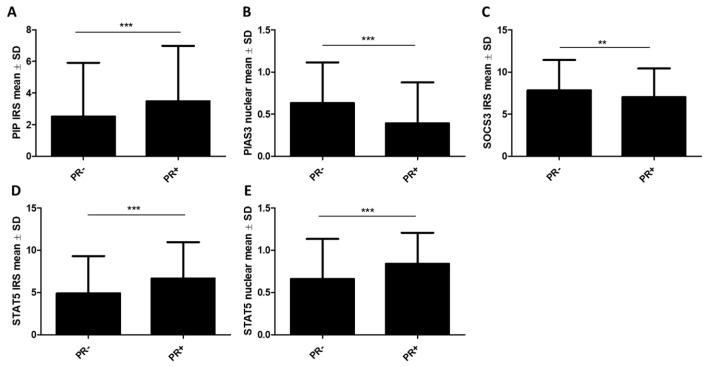
Protein expression levels of (**A**) PIP, (**B**) PIAS3 (nuclear), (**C**) SOCS3, (**D**) STAT5 (cytoplasmic), and (**E**) STAT5 (nuclear) according to the status of progesterone receptors (PR) (PR− *n* = 194, PR+ *n* = 360). ** *p* < 0.01, *** *p* < 0.001, Mann–Whitney U test. IRS, immunoreactive score; PIAS3, protein inhibitor of activated STAT3; PIP, prolactin-induced protein; PR, progesterone receptor; SD, standard deviation; SOCS3, suppressor of cytokine signaling 3; STAT5, signal transducer and activator of transcription 5.

Moreover, using the Mann–Whitney U test, the significance of differences in the expressions of the studied proteins was examined concerning ER, PR, and HER status. The study confirmed our previous results, showing higher immunoexpression levels of the PIP protein in ER+ (*n* = 410) cases (ER− vs. ER+: 2.59 ± 3.44 vs. 3.41 ± 3.5, *p* < 0.001) (Figure 4A), as well as in PR+ (*n* = 360) cases (PR- vs. PR+: 2.51 ± 3.41 vs. 3.48 ± 3.51, *p* < 0.001) (Figure 5A). Similarly, a higher expression of the STAT5 protein was noted in the group of ER+ tumors, for STAT5 (cytoplasmic) (ER− vs. ER+: 4.77 ± 4.38 vs. 6.66 ± 4.32, *p* < 0.001) (Figure 4E) and STAT5 (nuclear) (ER− vs. ER+: 0.64 ± 0.48 vs. 0.84 ± 0.37, *p* < 0.001) (Figure 4F), and in PR+ tumors for STAT5 (cytoplasmic) (PR− vs. PR+: 4.89 ± 4.34 vs. 6.66 ± 4.3, *p* < 0.001) (Figure 5D) and STAT5 (nuclear) (PR− vs. PR+: 0.65 ± 0.47 vs. 0.83 ± 0.36, *p* < 0.001) (Figure 5E). On the other hand, a reverse tendency was observed in the group of ER+ tumors in the case of PIAS3 (cytoplasmic) (ER− vs. ER+: 7.88 ± 3.44 vs. 6.99 ± 3.46, *p* = 0.0062) (Figure 4B), PIAS3 (nuclear) (ER− vs. ER+: 0.61 ± 0.49 vs. 0.41 ± 0.49, *p* < 0.001) (Figure 4C), SOCS3 (ER− vs. ER+: 7.77 ± 3.68 vs. 7.05 ± 3.4, *p* = 0.0092) (Figure 4D), and PRLR (ER− vs. ER+: 7.04 ± 2.9 vs. 5.75 ± 2.71, *p* = 0.0048) (Figure 4G), and in PR+ tumors in the case of PIAS3 (nuclear) (PR− vs. PR+: 0.63 ± 0.48 vs. 0.39 ± 0.49, *p* < 0.001) (Figure 5B) and SOCS3 (PR− vs. PR+: 7.82 ± 3.61 vs. 7 ± 3.43, *p* = 0.0026) (Figure 5C). There were no statistically significant differences in PRLR expression levels based on PR status, and no statistically significant differences in STAT5-P expression levels related to both hormonal receptors, ER and PR.

Only PRLR was significantly higher in HER2+ cases (*n* = 211) (HER2− vs. HER2+: 5.67 ± 2.65 vs. 6.49 ± 2.9, *p* = 0.027). The remaining antigens did not show statistically significant differences for HER2.

**Figure 6:**
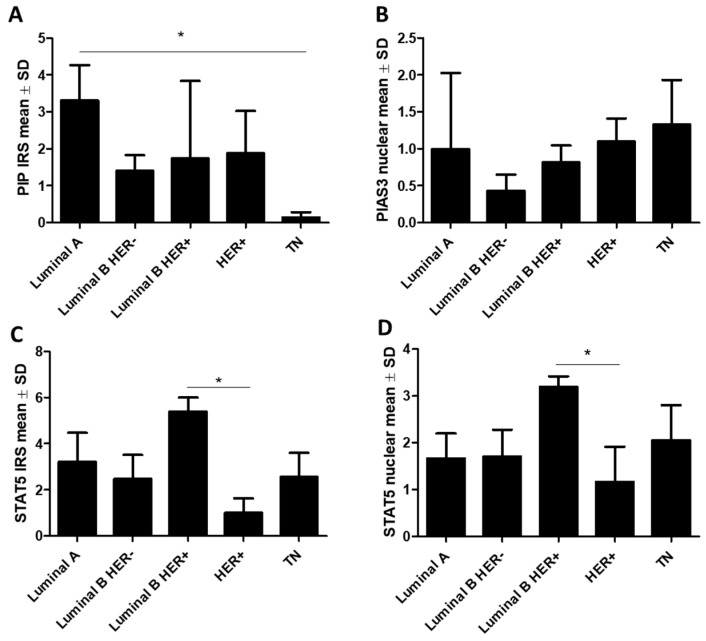
Expression of (**A**) PIP, (**B**) PIAS3 (nuclear), (**C**) STAT5 (cytoplasmic), and (**D**) STAT5 (nuclear) in molecular subtypes of BC (luminal A *n* = 145, luminal B HER− *n* = 143, luminal B HER+ *n* = 144, HER+ *n* = 67, TN *n* = 55). * *p* < 0.05, Kruskal–Wallis test with Dunn’s multiple comparison test. IRS, immunoreactive score; HER, human epidermal growth factor receptor; PIAS3, protein inhibitor of activated STAT3; PIP, prolactin-induced protein; SD, standard deviation; STAT5, signal transducer and activator of transcription 5; TN, triple negative breast cancer.

Differences in the protein levels (mean ± SD) among molecular types of breast cancer were examined using the Kruskal–Wallis test with Dunn’s multiple comparison test. Based on the molecular subtypes of BC, a significantly (*p* < 0.01) lower PIP immunoexpression level was observed in triple-negative (TN) cases (*n* = 55) (0.14 ± 0.38), and the highest in luminal A cases (*n* = 145) (3.31 ± 3.34) (luminal A vs. TN, *p* < 0.05) (Figure 6A). The opposite tendency was noted for PIAS3 (nuclear) (*p* = 0.07) (Figure 6B). The STAT5 expression level was the highest in luminal B HER+ (*n* = 144) (cytoplasmic: 5.38 ± 3.59 and nuclear: 3.2 ± 1.31) and the lowest in the HER+ type (*n* = 67) (cytoplasmic: 1.0 ± 1.55 and nuclear:1.17 ± 1.84; luminal B HER+ vs. HER+, *p* < 0.05) (Figure 6C,D). We did not observe any statistically significant differences in SOCS3, PIAS3 (cytoplasmic), STAT5-P, and PRLR expression according to the molecular types of BC. 

In patients with lymph node (N) metastases N+ (*n* = 227), a lower PIP level (N0 vs. N+: 3.38 ± 3.62 vs. 2.66 ± 3.209, *p* = 0.048) was demonstrated. No significant differences were noted for PIAS3 (cytoplasmic and nuclear) SOCS3, STAT5 (cytoplasmic and nuclear), STAT5-P, or PRLR.

**Figure 7:**
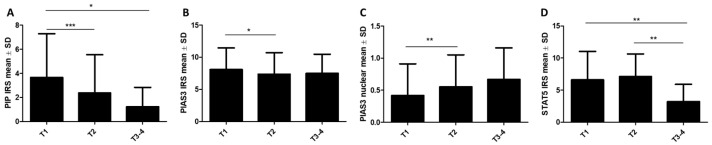
Differences in immunohistochemical levels of (**A**) PIP, (**B**) PIAS3 (cytoplasmic), (**C**) PIAS3 (nuclear), and (**D**) STAT5 (cytoplasmic) related to tumor size (T1 *n* = 255, T2 *n* = 272, T3–4 *n* = 27). * *p* < 0.05, ** *p* < 0.01, *** *p* < 0.001, Kruskal–Wallis test with Dunn’s multiple comparison test. IRS, immunoreactive score; PIAS3, protein inhibitor of activated STAT3; PIP, prolactin-induced protein; SD, standard deviation; STAT5, signal transducer and activator of transcription 5; T, tumor size.

Using the Kruskal–Wallis test with Dunn’s multiple comparison test, differences in the expression levels of the selected proteins regarding tumor size (T) and tumor/node/metastasis (TNM) clinical stages of cancer were demonstrated. In tumors classified as pT3–4 (*n* = 27), lower levels of PIP (T1: 3.66 ± 3.64, T2: 2.38 ± 3.18, T3–4: 1.23 ± 1.6, *p* < 0.001; T1 vs. T2: *p* < 0.001, T1 vs. T3–4: *p* < 0.05) (Figure 7A) and cytoplasmic STAT5 (T1: 6.6 ± 4.43, T2: 7.13 ± 3.51, T3–4: 3.2 ± 2.7, *p* = 0.019; T1 vs. T3–4: *p* < 0.01, T2 vs. T3–4, *p* < 0.01) (Figure 7D) were observed, while higher levels of nuclear PIAS3 (T1: 0.41 ± 0.49, T2: 0.55 ± 0.49, T3–4: 0.66 ± 0.49, *p* < 0.001; T1 vs. T2: *p* < 0.01) (Figure 7C) were noted. In the case of cytoplasmic PIAS3, a significant difference was noted between T1 and T2 size (T1: 8.09 ± 3.35, T2: 7.38 ± 3.33, T3: 7.49 ± 2.98, T1 vs. T2; *p* < 0.05) (Figure 7B). There were no significant differences concerning tumor size in SOCS3, PRLR, and STAT5-P immunoexpression. 

**Figure 8:**
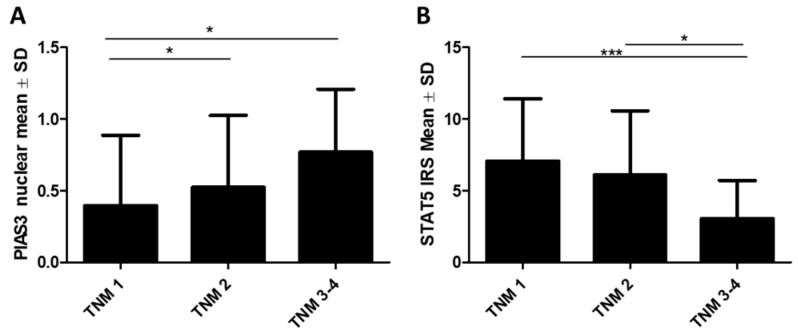
Protein expression levels of (**A**) PIAS3 (nuclear) and (**B**) STAT5 (cytoplasmic) with increasing TNM stages (TNM1 *n* = 161, TNM2 *n* = 238, TNM3–4 *n* = 155). * *p* < 0.05, *** *p* < 0.001, Kruskal–Wallis test with Dunn’s multiple comparison test. IRS, immunoreactive score; PIAS3, protein inhibitor of activated STAT3; SD, standard deviation; STAT5, signal transducer and activator of transcription 5; TNM, tumor/nodes/metastasis clinical stage.

No statistically significant differences in PIP, SOCS3, PIAS3 (cytoplasm), STAT5 (nucleus), STAT5-P, or PRLR levels were noted with TNM stage. In the case of the nuclear expression of PIAS3, an increase in its level with TNM stage was observed (TNM 1: 0.4 ± 0.49, TNM 2: 0.52 ± 0.5, TNM 3: 0.76 ± 0.43, *p* = 0.0035; TNM 1 vs. TNM 2, *p* < 0.05, TNM 1 vs. TNM 3–4, *p* < 0.05), as shown in Figure 8A, while a decrease in the cytoplasmic level of STAT5 was noted (TNM 1: 7.1 ± 4.3, TNM 2: 6.12 ± 4.47, TNM 3: 3.1 ± 2.66, *p* = 0.0004; TNM 1 vs. TNM 3–4, *p* < 0.001, TNM 2 vs. TNM 3–4, *p* < 0.05), as illustrated in Figure 8B. 

#### 2.1.2. Association of STAT Inhibitors (PIAS3 and SOCS3), STAT5, and PRLR with PIP Expression 

**Figure 9:**
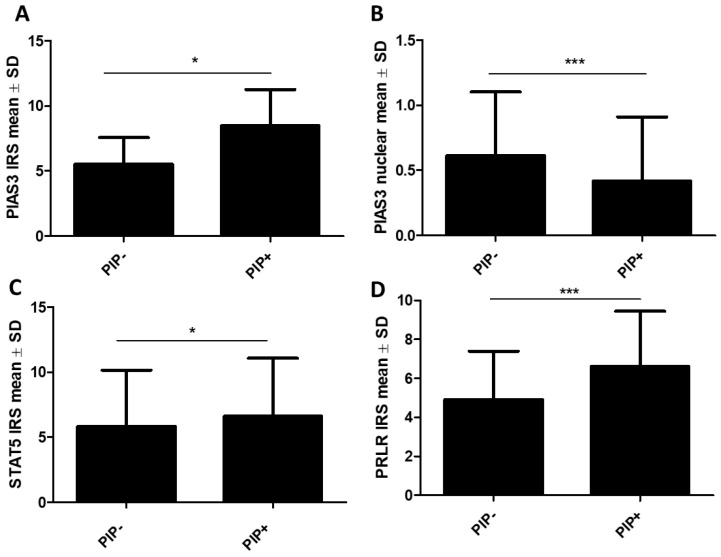
Protein expression levels of (**A**) PIAS3 (cytoplasmic), (**B**) PIAS3 (nuclear), (**C**) STAT5 (cytoplasmic), and (**D**) PRLR in PIP-positive (PIP+ *n* = 377) and PIP-negative cases (PIP− *n* = 177). * *p* < 0.05, *** *p* < 0.001, Mann–Whitney U test. IRS, immunoreactive score; PIAS3, protein inhibitor of activated STAT3; PIP, prolactin-induced protein; PRLR, prolactin receptor; SD, standard deviation; STAT5, signal transducer and activator of transcription 5.

Using the Mann–Whitney U test, we observed higher levels of cytoplasmic PIAS3 (PIP− vs. PIP+: 5.5 ± 2.1 vs. 8.5 ± 2.79, *p* = 0.018) (Figure 9A) and significantly lower nuclear PIAS3 (PIP− vs. PIP+: 0.61 ± 0.49 vs. 0.42 ± 0.49, *p* = 0.0006) in PIP-positive cases (*n* = 377), (Figure 9B). Additionally, cytoplasmic STAT5 (PIP− vs. PIP+: 5.82 ± 4.33 vs. 6.6 ± 4.46, *p* = 0.047) expression (Figure 9C), as well as PRLR (PIP− vs. PIP+: 4.9 ± 2.49 vs. 6.61 ± 2.83, *p* < 0.001), were significantly higher in PIP+ (Figure 9D). We did not observe significant differences in positive and negative PIP cases (*n* = 177) for SOCS3, STAT5 (nuclear), or STAT5-P.

#### 2.1.3. Associations with Survival

**Figure 10:**
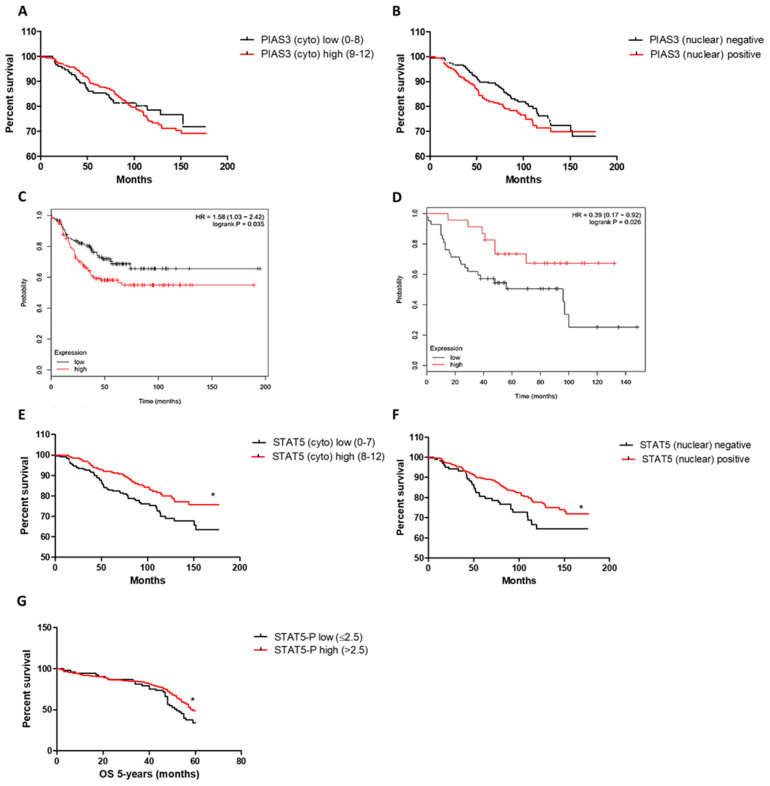
Associations with patients’ overall survival (OS) of: (**A**) PIAS3 (cytoplasmic) (PIAS3 (cyto) low (0–8) *n* = 388, PIAS3 (cyto) high (9–12) *n* = 166; cut-off, median), (**B**) PIAS3 (nuclear) (PIAS3 (nuclear) negative *n* = 255, PIAS3 (nuclear) positive *n* = 299), (**C**) *PIAS3* mRNA (Kaplan–Meier Plot [34]) in BC patients (*PIAS3* low *n* = 131, *PIAS3* high *n* = 124; cut-off, median), (**D**) *PIAS3* mRNA (Kaplan–Meier Plot [34]) in TN BC patients (*PIAS3* low *n* = 42, *PIAS3* high *n* = 23; cut-off, median), (**E**) STAT5 (cytoplasmic) (STAT5 (cyto) low (0–7) *n* = 294, STAT5 (cyto) high (9–12) *n* = 260; cut-off, median), (**F**) STAT5 (nuclear) (STAT5 (nuclear) negative *n* = 117, STAT5 (nuclear) positive *n* = 437), and (**G**) association of the STAT5-P with 5-year-OS (STAT5-P low (≤2.5) *n* = 238, STAT5-P high (>2.5) *n* = 316; cut-off, median). * *p* < 0.05; BC, breast cancer; PIAS3, protein inhibitor of activated STAT3; STAT5, signal transducer and activator of transcription 5; STAT5-P, phosphorylated signal transducer and activator of transcription 5; TN, triple negative.

Survival analysis showed that a higher PIAS3 expression in all examined cases was associated with longer overall patient survival (OS), but only with an observation time shorter than 100 months(median overall survival (OS): not reached; hazard ratio (HR) = 1.01, 95% CI 0.29–3.87) (Table 1A). Over this time, we observed an inverse trend, as shown in Figure 10A. Moreover, patients with a lack of PIAS3 in the nucleus were characterized by longer patient survival (mOS: not reached, HR = 3.41, 95% CI 0.86–13.6) (Table 1A), as shown in Figure 10B. Kaplan–Meier plots based on mRNA showed that a higher level of *PIAS3* mRNA was related to a shorter OS survival of BC patients (mOS: not reached, HR = 1.58, 95% CI 1.03–2.42, *p* = 0.035) (Figure 10C). However, in TN cases, an inverse situation was noted (mOS low: 49, mOS high: not reached, HR = 0.39, 95% CI 0.17–0.92 *p* = 0.03), as shown in Figure 10D. Additionally, in the case of STAT5 (cytoplasmic and nuclear) (Figure 10E,F), higher expressions of this protein were related to better outcomes (mOS: not reached, cytoplasmic: HR = 0.5, 95% CI 0.11–2.35, *p* = 0.032; nuclear: HR = 0.36, 95% CI 0.07–1.95, *p* = 0.011) (Table 1A). A higher STAT5-P expression was characteristic of a longer 5-year OS for patients with BC (mOS low: 52, mOS high: 59, HR = 0.59, 95% CI 0.44–2.43) (Figure 10G). SOCS3 and PRLR did not significantly correlate with patients’ survival. Moreover, multivariate Cox survival analyses showed that only HER2 was an independent prognostic factor in the case of DSF (HR = 2.01, 95% CI 1.18–3.43, *p* = 0.011) (Table 1B). The median overall survival (mOS) and disease-free survival (mDFS) for clinicopathological factors are described in Table S1.

### 2.2. Real-Time qPCR

#### 2.2.1. RT-qPCR Detection of PIAS3, SOCS3, STAT5, and PIP Gene Expression in BC and Control Tissues

**Figure 11:**
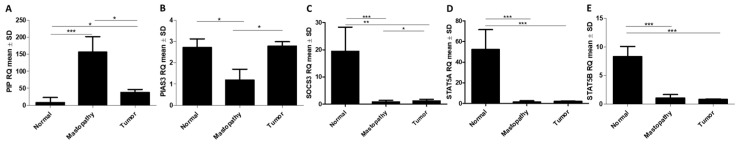
Relative mRNA expression levels (RQ) of (**A**) *PIP*, (**B**) *PIAS3*, (**C**) *SOCS3*, (**D**) *STAT5A*, and (**E**) *STAT5B* in BC tissue (*n* = 42) in comparison to normal tissue (margin) (*n* = 40) and mastopathy (*n* = 16). * *p* < 0.05, ** *p* < 0.01, *** *p* < 0.001, Kruskal–Wallis test with Dunn’s multiple comparison test. PIAS3, protein inhibitor of activated STAT3; PIP, prolactin-induced protein; SD, standard deviation; SOCS3, suppressor of cytokine signaling 3; STAT5A, signal transducer and activator of transcription 5A; STAT5B, signal transducer and activator of transcription 5B; STAT5-P, phosphorylated signal transducer and activator of transcription 5.

Using RT-PCR, the expression levels of *PIP, PIAS3, SOCS3, STAT5A,* and *STAT5B* were verified at the level of mRNA isolated from the tumor’s margin (normal) (*n* = 40), mastopathy (*n* = 16), and BC tumor tissue (*n* = 42). The mean relative quantification (RQ) ± SD mRNA level of analyzed genes in the tissue material of BC was, respectively, *PIP* (57.37 ± 98.92), *PIAS3* (3.07 ± 1.6), *SOCS3* (1.25 ± 0.87), *STAT5A* (2.14 ± 1.86), and *STAT5B* (0.8 ± 0.54). Using the Kruskal–Wallis test with Dunn’s multiple comparisons, we demonstrated a difference in mRNA levels in tumors compared to controls with tissues from the tumor margin (normal) and mastopathy. *PIP* expression was significantly lower in the normal tissue in comparison to mastopathy (normal vs. mastopathy: 8.06 ± 14.91 vs. 156.9 ± 151.9, *p* < 0.001) and to tumors (normal vs. tumor: 8.06 ± 14.91 vs. 37.78 ± 53.02, *p* < 0.05). The mean *PIP* mRNA RQ level in tumors was lower than that in mastopathy (tumor vs. mastopathy: 37.78 ± 53.02 vs. 156.9 ± 151.9, *p* < 0.05) (Figure 11A). On the other hand, in the case of *PIAS3* (*p* = 0.0091)*, SOCS3* (*p* < 0.001), *STAT5A*, and *STAT5B*, there was an inverse trend indicating a much higher mRNA level (RQ mean ± SD) in normal tissue compared to mastopathy (normal vs. mastopathy: *PIAS3* 2.72 ± 2.34 vs. 1.19 ± 0.51, *p* < 0.05; *SOCS3* 19.46 ± 52.61 vs. 0.78 ± 0.57, *p* < 0.001; *STAT5A* 52.24 ± 112.4 vs. 1.42 ± 1.27, *p* < 0.001; *STAT5B* 8.28 ± 10.47 vs. 1.04 ± 0.61, *p* < 0.001) (Figure 11B–E). Interestingly, we noted that the expressions of *PIAS3* (*p* = 0.0091) and *SOCS3* (*p* < 0.05) were higher in tumors in comparison to mastopathy (tumor vs. mastopathy: *PIAS3* 2.79 ± 1.26 vs. 1.19 ± 0.51, *p* < 0.05; *SOCS3* 1.15 ± 0.64 vs. 0.78 ± 0.57, *p* < 0.05), and *STAT5* was significantly lower in tumors in comparison to the tumor margin (tumor vs. normal: *STAT5A* 2.14 ± 1.86 vs. 52.24 ± 112.4, *p* < 0.001; *STAT5B* 0.8 ± 0.54 vs. 8.28 ± 10.47, *p* < 0.001). There was no significant difference in *STAT5A* and *STAT5B* expression in BC compared to mastopathy.

Moreover, the obtained results demonstrated a statistically significant decreased level (RQ mean ± SD) of *PIP* expression (G1: 149.5 ± 103, G2: 39.55 ± 31.63, G3: 14.94 ± 32, *p* < 0.001) with an increasing histological grade of the tumors (G) (G1 *n* = 7, G2 *n* = 24, G3 *n* = 11). In the case of *PIAS3*, *SOCS3*, *STAT5A,* and *STAT5B,* there were no significant relationships with G at the mRNA level.

**Figure 12:**
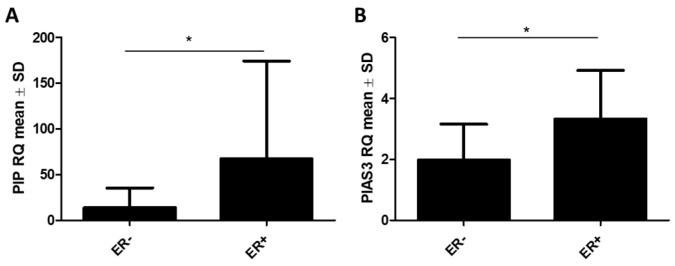
mRNA levels of (**A**) *PIP* and (**B**) *PIAS3* according to the status of estrogen receptors (ER) (ER− *n* = 10, ER+ *n* = 32). * *p* < 0.01, Mann–Whitney U test. ER, estrogen receptor; PIAS3, protein inhibitor of activated STAT3; PIP, prolactin-induced protein; SD, standard deviation.

Additionally, we observed higher *PIP* mRNA levels in ER+ cases (ER− vs. ER+: 13.7 ± 21.5 vs. 67.24 ± 106.9, *p* = 0.0384) (Figure 12A). Similarly, a higher expression of *PIAS3* (ER− vs. ER+: 1.97 ± 1.18 vs. 3.33 ± 1.6, *p* = 0.03) was noted in the group of ER+ tumors (*n* = 32) (Figure 12B). No other positive relations with clinicopathological factors (G, PR, pN, TNM, and HER2) were observed at the mRNA level for *PIP, PIAS3, SOCS3*, *STAT5A*, and *STAT5B*.

#### 2.2.2. Correlations Between PIP, PIAS3, SOCS3, STAT5, and PRLR at Protein and mRNA Level

**Figure 13:**
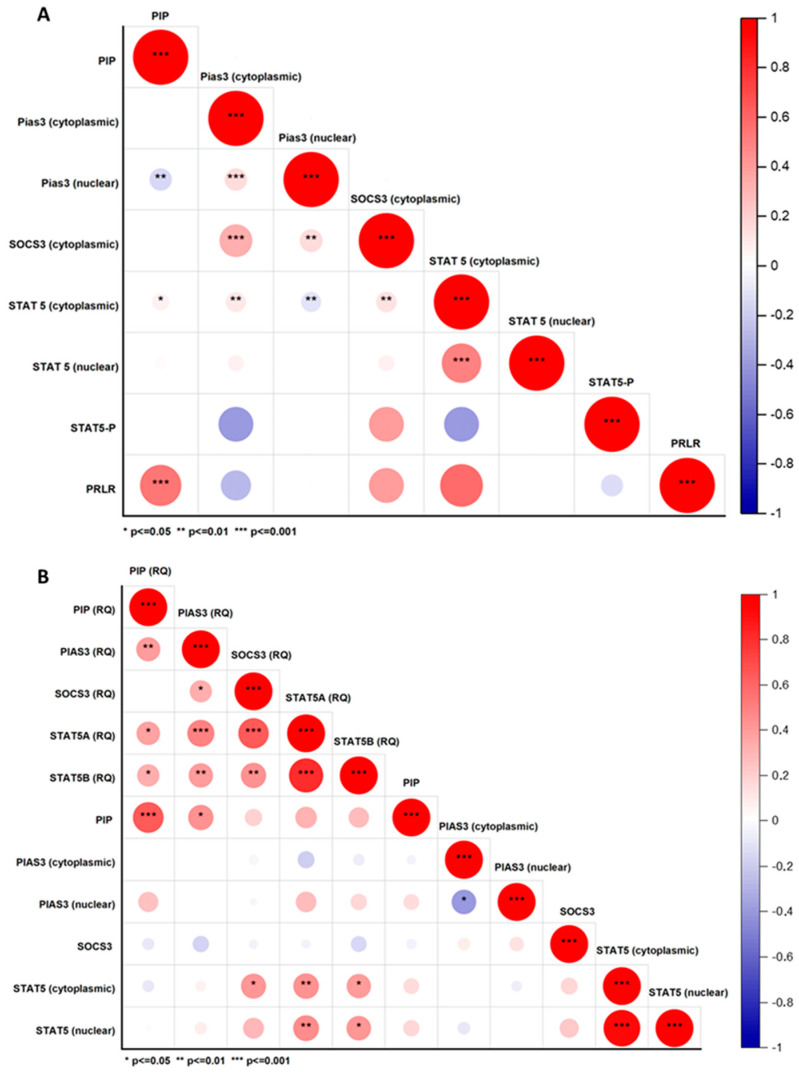
Spearman rank correlation matrix for studied molecules PIP, PIAS3, SOCS3, STAT5, and PRLR at (**A**) protein (*n* = 554) and (**B**) mRNA levels (*n* = 42). Positive correlations are shown in red, and negative correlations in blue. The circle’s size and color intensity correspond to the correlation coefficients. The correlation coefficients and matching colors are displayed in the color bar on the right side of the graphic. * *p* ≤ 0.05, ** *p* ≤ 0.01, *** *p* ≤ 0.001, Spearman rank correlations. PIAS3, protein inhibitor of activated STAT3; PIP, prolactin-induced protein; PRLR, prolactin receptor; RQ, relative quantification, real-time PCR; SOCS3, suppressor of cytokine signaling 3; STAT5, signal transducer and activator of transcription 5; STAT5-P, phosphorylated signal transducer and activator of transcription 5.

Spearman’s correlations of the tested molecules were assessed based on an evaluation of the immunohistochemical and RT PCR reactions. A high positive correlation for PIP and *PIP* (RQ) was noted (*r* = 0.66, *p* ≤ 0.001), as well as for PIP and PRLR (*r* = 0.56, *p* ≤ 0.001). There was a statistically significant low negative correlation for PIP and PIAS3 (nuclear) (*r* = −0.18, *p* ≤ 0.01) and a low positive correlation for PIP and STAT5 (cytoplasmic) (*r* = 0.1, *p* ≤ 0.01). Additionally, a medium positive correlation for *PIP* (RQ) and *PIAS3* (RQ) (*r* = 0.41, *p* < 0.05), *STAT5A* (RQ) (*r* = 0.38, *p* ≤ 0.05), and *STAT5B* (RQ) (*r* = 0.35, *p* ≤ 0.05) was observed. There were no significant correlations between PIP and SOCS3 at both the protein and mRNA levels. All significant correlations of the studied molecules are presented in correlation plots (Figure 13). 

Only PIAS3 showed significant differences and correlations with PIP among the studied STAT inhibitors. Consequently, studies of PIAS3 were undertaken on the T47D cell line, which is characterized by a high expression of PIP and the presence of PRLR. To confirm the potential influence of PIAS3 expression on the PIP level, PIAS3 was silenced in the T47D cell line (T47D shPIAS3) using a lentiviral vector. The effect of PIAS3 silencing in T47D shPIAS3 cells was measured by IF, RT-PCR, and WB. The above methods were also used to investigate changes in the expression levels of PIP, STAT5, STAT5-P, and PRLR. Since PIP is a prolactin-induced protein, changes in the expression level of PIP were also analyzed after the incubation of T47D cells with recombinant human PRL. In addition, the effect of PIAS3 silencing on cell viability and their sensitivity to PRL, doxorubicin (DOX), and a combination of both compounds was measured. 

### 2.3. Cell Lines (In Vitro Tests)

#### 2.3.1. Assessment of Protein Localization and Fluorescence Intensity in the T47D Cell Lines

**Figure 14:**
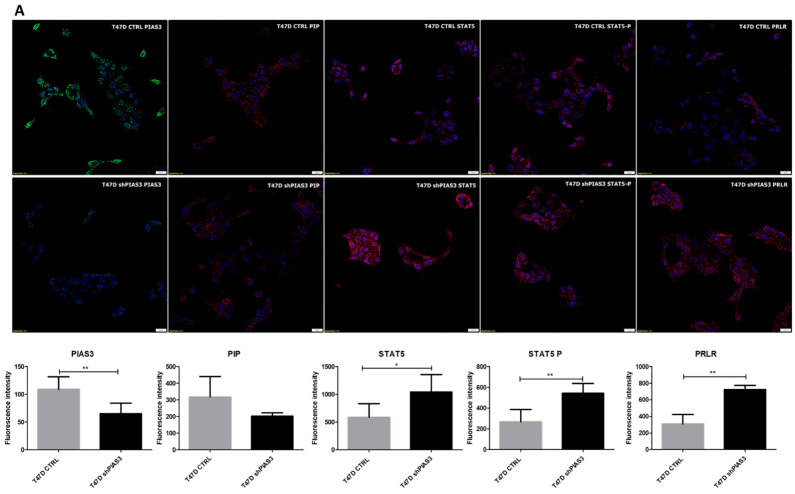

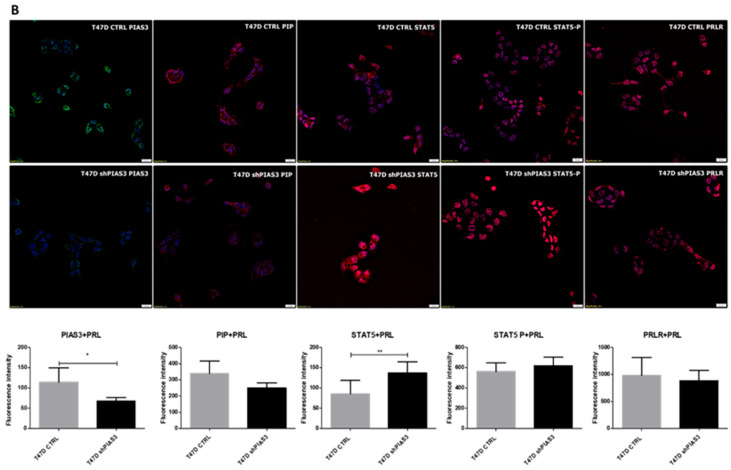
Confocal microscope images demonstrating the localization and fluorescence intensity of PIP, PIAS3, SOCS3, STAT5, and PRLR in T47D CTRL and T47D shPIAS3 (**A**) without PRL and (**B**) after incubation with 1 ng/mL PRL * *p* < 0.05, ** *p* < 0.01, Mann–Whitney U test. PIAS3, protein inhibitor of activated STAT3; PIP, prolactin-induced protein; PRL, prolactin; PRLR, prolactin receptor; SOCS3, suppressor of cytokine signaling 3; STAT5, signal transducer and activator of transcription 5; STAT5-P, phosphorylated signal transducer and activator of transcription 5.

To verify the localization and expression levels of PIP, PIAS3, SOCS3, STAT5, and PRLR, immunofluorescence (IF) was performed on the T47D cell line, with control (T47D CTRL) and silenced PIAS3 (T47D shPIAS3) groups. Analysis of the IF results indicated the cytoplasmic localization of PIP and PIAS3, membranous localization of PRLR, and both cytoplasmic and nuclear localization in the case of STAT5 and STAT5-P (Figure 14A). PIAS3 silencing significantly increased the level of the STAT5 protein and its phosphorylated form. An increased level of PRLR was observed in the T47D shPIAS3 cell line. Preincubation (20 min) with 1 ng/mL of recombinant human PRL significantly increased STAT5 levels (both STAT5 and STAT5-P), resulting in their nuclear translocation compared to the control cell line (Figure 14B). There was no significant difference in PIP level in the T47D shPIAS3 line compared to the control without and after incubation with PRL.

#### 2.3.2. RT-PCR Analysis of mRNA Expression Levels of PIAS3, PIP, STAT5A, and STAT5B in T47D Cell Lines

The significance of the MANOVA model was tested with four different statistical tests to measure the effect of PRL, DOX, and PRL+DOX on the mRNA levels of the tested markers in T47D shPIAS3 and T47D CTRL cells. The results of multivariate tests such as Wilks’ Lambda, Pillai’s Trace, Hotelling’s Trace, and Roy’s Largest Root showed statistically significant differences between the studied markers (*p* < 0.05 for each test), as shown in Table S2. The Bonferroni post hoc test was also performed to assess which dependent variables differed significantly between groups. 

**Figure 15:**
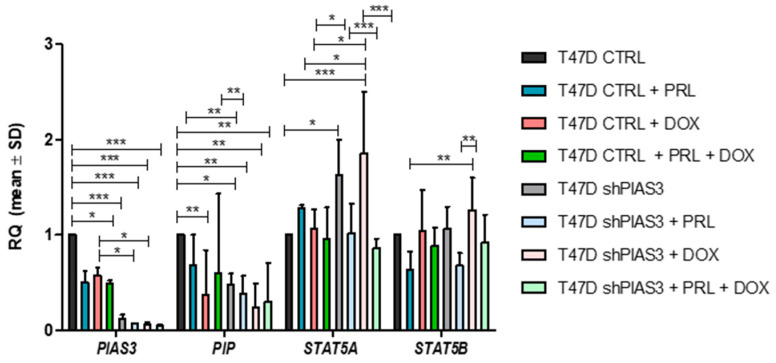
Relative mRNA expression levels (RQ) of *PIAS3, PIP, STAT5A*, and *STAT5B* in T47D cell lines: control T47D CTRL vs. PIAS3-silenced T47D shPIAS3. The graph shows the level of mRNA isolated from cells cultured in medium (RPMI), after incubation with PRL (1 ng/mL, 20 min), DOX (0.5 µM, 48 h), and with both: PRL (1 ng/mL, 20 min) and DOX (0.5 µM, 48 h). Data represent the mean ± SD of three independent measurements. * *p* < 0.05, ** *p* < 0.01, *** *p* < 0.001; two-way MANOVA with Bonferroni post hoc tests. DOX, doxorubicin; PIAS3, protein inhibitor of activated STAT3; PIP, prolactin-induced protein; PRL, prolactin; PRLR, prolactin receptor; RQ, relative quantification, real-time PCR; SD, standard deviation; SOCS3, suppressor of cytokine signaling 3; STAT5A, signal transducer and activator of transcription 5A; STAT5B, signal transducer and activator of transcription 5B; STAT5-P, phosphorylated signal transducer and activator of transcription 5.

The PCR results showed a significant decrease in *PIAS3* mRNA levels in T47D shPIAS3 cells to the control T47D CTRL cells (*p* < 0.001). PIAS3 silencing in the T47D shPIAS3 line decreased *PIP* mRNA levels compared to the control T47D CTRL cells (*p* < 0.05). After adding PRL or DOX alone, a downward trend in *PIP* mRNA expression was observed (*p* < 0.01). Silencing the STAT5 inhibitor (PIAS3) in the T47DshPIAS3 line increased *STAT5A* levels compared to the control T47D CTRL cells (*p* < 0.01). PIAS3 silencing did not cause changes in *STAT5B* levels. When PRL was added alone, the *STAT5A* levels increased in T47D CTRL cells. DOX alone increased *STAT5A* (*p* < 0.01) and *STAT5B* levels in the T47D shPIAS3 cell line (Figure 15).

#### 2.3.3. Western Blot Analysis of Cytoplasmic and Nuclear Fractions Isolated from T47D Cell Lines

**Figure 16:**
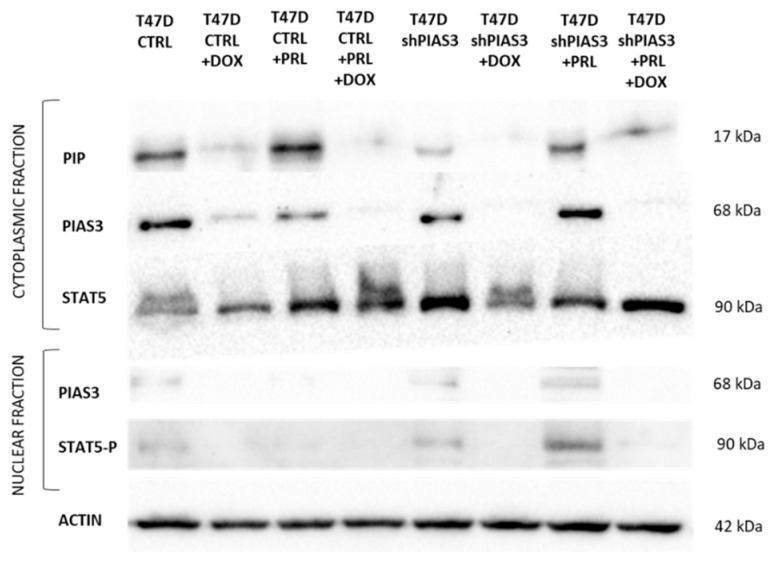
Western blot analysis of cytoplasmic and nuclear fractions of PIP, PIAS3, STAT5, and STAT5-P in T47D cell lines: control T47D CTRL vs. PIAS3-silenced T47D shPIAS3. The figure shows the level of protein isolated from cells cultured in medium (RPMI), after incubation with PRL (1 ng/mL, 20 min), DOX (0.5 µM, 48 h), and both: PRL (1 ng/mL, 20 min) with DOX (0.5 µM, 48 h). DOX, doxorubicin; PIAS3, protein inhibitor of activated STAT3; PIP, prolactin-induced protein; PRL, prolactin; PRLR, prolactin receptor; SOCS3, suppressor of cytokine signaling 3; STAT5, signal transducer and activator of transcription 5; STAT5-P, phosphorylated signal transducer and activator of transcription 5.

Western blot analysis of protein lysates from T47D CTRL and T47D shPIAS3 cell lines confirmed the RT-PCR data and reflected the results at the mRNA level. Proteins were isolated from cytoplasmic and nuclear fractions. The level of PIP protein in the line with silenced PIAS3 was lower than that in the control. The addition of PRL had a stimulatory effect on the level of PIP protein in both the T47D CTRL and T47D shPIAS3 cell lines. An increased STAT5 level was visible in T47D shPIAS3 cells both when untreated and after incubation with PRL compared to T47D CTRL cells (Figure 16). 

#### 2.3.4. MTT (3-(4,5-Dimethylthiazol-2-yl)-2,5-Diphenyltetrazolium Bromide)—Cell Viability Assay

**Figure 17:**
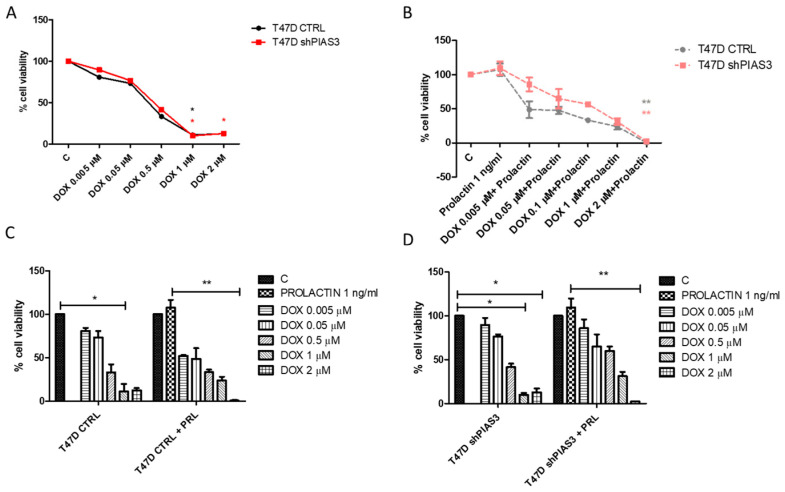
MTT analysis of cell viability and cytotoxic effect of increasing doses of (**A**) DOX and (**B**) DOX with PRL (1 ng/mL) on T47D CTRL and T47D shPIAS3 cell line. Following preincubation with PRL, T47D CTRL cells exhibited greater sensitivity to DOX than the T47D shPIAS3 cell line. Incubation with PRL increased cell viability of (**C**) T47D CTRL and (**D**) T47D shPIAS3. (**A**,**B**) (significant differences vs. control (**C**)); * *p* < 0.05, ** *p* < 0.01, Kruskal–Wallis test with Dunn’s multiple comparison test. Data represent the mean ± SD (standard deviation) of eight replicates from three independent measurements. DOX, doxorubicin; PIAS3, protein inhibitor of activated STAT3; PRL, prolactin.

PIAS3 silencing had no significant effect on the sensitivity of T47D cells to DOX (Figure 17A). The T47D CTRL line (with a higher PIP expression) after preincubation with PRL was more sensitive to DOX compared to T47D shPIAS3 cells (Figure 17B). Incubation with 1 ng/mL of PRL for 20 min increased the cell viability of T47D CTRL and T47D shPIAS3 (Figure 17C,D).

## 3. Discussion

Cancer is currently the most common cause of death worldwide. One of the primary goals in the era of personalized medicine is to search for new prognostic biomarkers. Identifying key signaling pathways and regulatory molecules, such as PIAS3, PIP, STAT5, and SOCS3 in breast cancer (BC) cells, can provide valuable insights into the mechanisms of the disease and potentially lead to the development of targeted therapies. The varying expressions of these molecules in response to stimuli, such as PRL and DOX, underscore their importance in cancer progression and treatment response. Understanding the complex interactions between these factors and their effects on cell viability and sensitivity to chemotherapy could lead to new approaches for personalized cancer therapy, ultimately improving patient outcomes. 

Previous studies have shown that PIP, PIAS3, SOCS3, and STAT5 are expressed differently in tumors at both the protein and gene levels [14,35,36,37,38]. Research on human BC has indicated that PIAS3 and SOCS3 play direct roles in regulating JAK/STAT signaling [33,39,40]. Although the roles of SOCS3 and PIAS3 in BC remain correlated, their relations with other factors, such as PIP, remain poorly understood. To our knowledge, this is the first paper describing the potential relationship of PIP with selected STAT5 inhibitors (SOCS3 and PIAS3). 

In our research, we noticed a negative correlation between PIP, STAT5, and the nuclear expression of PIAS3. The PIAS family can modulate the JAK/STAT pathway in different ways, like masking the DNA-binding domain of STAT or binding to STATs, preventing their dimerization [37]. The inverse correlation between cytoplasmic STAT5 and nuclear PIAS3 may indicate its translocation from the cytoplasm to the cell nucleus, suggesting its participation in the organization of chromatin. Due to the SAP domain, PIAS3 plays a role in chromatin biology by recruiting histone deacetylases to repress the transcriptional activity of STAT proteins [37]. Additionally, PIAS proteins participate in the relocation of transcriptional regulators to various subnuclear compartments [41]. This may be important in regulating PIP expression, considering the negative correlation of nuclear PIAS3 with the PIP protein. Interestingly, we found a moderate positive correlation between PIP and PIAS3 at the mRNA level. The PIAS3 protein might indirectly regulate PIP expression as E3 ligases for the small ubiquitin-like modifier (SUMO) modification pathway [41]. This was demonstrated for proteins such as LEF1, p53, and the AR. AR expression is strongly correlated with PIP and bound to androgen-responsive elements of the PIP promoter [7,42]. The effect of PIAS proteins is difficult to predict. On the one hand, PIASy can inhibit AR activity, while PIAS3 can act as a stimulator of the AR under the same conditions [43]. PIAS3 can act as either an activator or repressor of STAT5, but its mechanism of action depends on the target gene and promoter context in which the PIAS3–STAT5 complex finds itself [44]. 

In our investigation, we also observed a positive correlation between PIP and STAT5 at the protein and mRNA levels. A higher level of STAT5 was noted in PIP-positive cases, which may be related to the upregulation of PIP through these factors. This aligns with previous findings that showed differences in *STAT5A* and *STAT5B* gene expression in PIP-expressing BC cells compared to PIP-negative cells [20]. There is evidence that PRL, through its receptor, induces the phosphorylation of STAT5A and/or STAT5B, which are translocated into the nucleus after dimerization. There, they bind to the STAT5-responsive element to increase the transcription of the PIP gene [45]. The JAK/STAT pathway is responsible for passing versatile signals that lead to inflammatory responses, differentiation, cell migration, apoptosis, and proliferation, which makes STATs an attractive target for oncogenic therapeutic intervention [37]. Blocking STAT5′s function alone has been demonstrated to be sufficient to inhibit tumor cell growth and induce apoptosis [46]. STAT3 and STAT5 are the most associated with human cancers and tumor cell lines in response to different oncogenic kinases [47]. The role of STAT5 in BC is a topic of intense debate, with conflicting evidence depending on the specific model being studied. Rat and human BC tissues display elevated nuclear STAT5A and increased nuclear levels of STAT DNA-binding activity [47]. The overexpression of STAT5A in T47D-derived breast tumors results in a reduced tumor size due to increased apoptosis [40]. This may be directly related to our previous findings, indicating that the increased sensitivity of BC cells to chemotherapy is due to PIP’s pro-apoptotic activity, as shown by the upregulation of specific pro-apoptotic genes [14]. 

Contrary to our assumptions, we did not find significant correlations between PIP and SOCS3. However, both tested STAT inhibitors, PIAS3 and SOCS3, showed correlations at the protein level. SOCS3 may act like a double-edged sword in regulating the molecular pathways of carcinogenesis [36]. The altered expression of SOCS3 in cancer cells is linked to the dysregulation of cell growth, migration, and apoptosis in human cancers [48]. Immunohistochemical analysis has shown the cytoplasmatic localization of SOCS3 [36]. Lines of evidence suggest that SOCS3 expression is higher in adjacent tissues compared to BC tumor tissues [49,50,51]. Our research showed a similar trend, with a higher SOCS3 mRNA expression in the tumor margin compared to neoplastic tissue. Additionally, we identified a positive correlation between SOCS3 and STAT5. This relationship is attributed to the inducible nature of SOCS proteins by cytokine stimulation, as they function within a classical negative feedback loop [48]. SOCS3 reduces STAT5 phosphorylation through its competitive action [40]. Pezet et al. showed that SOCS proteins have dual effects, inhibiting and restoring PRL signaling through different mechanisms [28]. Importantly, despite the constitutively phosphorylated STATs in T47D, they do not bind to the SOCS3 promotor without PRL treatment. Authors have suggested that PRL stimulates SOCS3 expression in a STAT5- and Sp1-dependent manner. The PRL-dependent recruitment of Sp1 to the SOCS3 promoter may be a prerequisite for STAT binding due to its ability to modify the chromatin structure and increase the availability of regulatory motifs [46]. SOCS3 inhibits STAT5 activation and subsequently decreases cell proliferation [40]. So far, data have shown associations of SOCS3 with benign tumors and indicate a decrease in SOCS3 levels as the tumor’s stage advances [36]. A lower expression of SOCS3 was correlated with poorly differentiated tumors [52]. Despite a similar trend, our results were not statistically significant at the protein and mRNA levels. According to a meta-analysis, SOCS3 expression was not associated with molecular subtype, tumor size, tumor stages, and lymph node invasion, which is consistent with our findings [52].

Growing evidence has revealed that PIP expression is correlated with apocrine differentiation, hormone receptor status, and longer metastasis-free survival. Moreover, PIP expression depends on the tumor stage and molecular subtype of BC [38]. According to previous studies, we noted a higher PIP expression in early-stage tumors of the luminal A subtype and the lowest in triple-negative subtypes, which have a poorer prognosis. These data are consistent with the results presented in earlier studies, which showed decreased PIP levels with an increasing histology grade [53,54]. STAT5 shows a similar expression profile, which may support previous evidence that it acts as a transcriptional factor upregulating PIP expression [20]. There is evidence that the activation of STAT5 is associated with well-differentiated BCs, less aggressiveness, and an increased sensitivity to chemotherapy for BC. In line with the results of Nevalainen et al., we noted a gradual decrease in STAT5 as the tumors advanced [55,56,57]. Consistent with these results, our study shows that a positive STAT5 expression and STAT5-P are significantly higher in mastopathy than in tumors. STAT5 may act as an oncogene during tumorigenesis and as a tumor suppressor in the early phase of the disease, gradually becoming inactivated with cancer progression [58,59]. 

Interestingly, the opposite trend was shown in the case of PIAS3. A low PIP immunoexpression level with a simultaneously high level of nuclear PIAS3 in G3-histological-grade and triple-negative cases may be considered as evidence suggesting a regulatory role of PIAS3 in PIP expression. We did not observe significant differences in cytoplasmic PIAS3 or PIAS3 mRNA levels between histological grades. This contrasts with Taheri et al.’s results, which demonstrated a higher PIAS3 gene level in G1-grade cases [60]. Recent studies have shown that the overexpression of PIAS3 in BC cell lines can significantly impact STAT5-mediated gene expression, such as PIP, and induce apoptosis [16]. The distribution of PIAS3 in BC cells is variable. Its cytoplasmic and/or nuclear localization is related to the type of interaction and function of the protein. As described in published studies, the relationship between PIAS3 and clinical data yields inconclusive results. Some authors revealed that the PIAS3 gene is significantly downregulated in BC compared to adjacent noncancerous tissues, indicating that lower PIAS3 levels in breast tissue may be linked to the development of BC [60]. In contrast, McHale et al. reported a higher level of PIAS3 in DCIS/IDC compared to normal/hyperplastic tissue [16]. Our studies indicated higher levels of PIAS3 mRNA in normal and tumor tissues and significantly lower levels in mastopathy. This observation may support the hypothesis that a decrease in PIAS3 expression level is associated with the initiation stage of the carcinogenesis process. The high level of PIAS3 mRNA in the normal tissues may suggest a protective function for the cell. The lower level of PIAS3 protein in tumors compared to mastopathy may be the effect of a potential post-translational modification of this protein or degradation in one of the abovementioned mechanisms. Furthermore, the positive correlation between PIAS3 and PIP at the mRNA level may suggest that PIAS3 participates in regulating PIP expression through a negative feedback loop [61].

The results of this study indicate that the expression of PIP is observed in various BC subtypes, with a higher prevalence in hormone-receptor-positive cases. We noted that both PIP and STAT5 immunoexpression were statistically higher in ER+ and PR+ patients, while the STAT inhibitors, PIAS3 and SOCS3, were lower in cases with a positive status of those receptors. These data are consistent with the results of Taheri et al., who stated that PIAS1–3 expressions were significantly lower in ER+ samples than in ER− ones [60]. PIAS3 may facilitate ER signaling as a SUMO-1 E3 ligase for ERα sumoylation [62]. This may suggest that the relationships of the studied proteins depend on the status of hormonal receptors and hormonal regulation.

STAT5 has been detected in all types of BC, in ER+, HER2+, and TN, and can act as a tumor suppressor or oncogene under different conditions [55,63]. Our results indicate that STAT5 expression level is related to luminal B HER+, suggesting the involvement of HER2 in the PRL signaling pathway. Kavarthapu et al. propose that resistance to endocrine therapy in BC patients may be associated with elevated levels of circulating PRL and an increased expression of HER2 [64,65,66]. The induction of the HER2 signaling pathway with PRL participation may influence the transcription of PRLR and other ER target genes. PRL via PRLR/JAK2 induces the phosphorylation of HER2, which activates the RAS/MEK/ERK signaling pathway required for ERα phosphorylation [65]. Activated ER forms a complex with Sp1/C/EBPβ and is recruited with STAT5 to the PRLR or/and the PIP promoter [20]. Björnström et al. demonstrated a cross-talk between STAT5 and the nuclear hormone receptors, including ERα and Erβ [67]. STAT5 can modulate ER transcriptional activity, potentially affecting BC cells’ responsiveness to hormonal therapies [66,67]. In ER+ patients, STAT5 expression enhanced the response to hormone therapy and increased the OS [63,68]. 

A study examining over 1300 BCs found a strong association between STAT5 nuclear localization/phosphorylation and an improved OS and DFS [68]. Based on our data, we found that STAT5 is linked to a better OS. Experiments on human BC cell lines indicate that STAT5′s capacity to encourage cellular differentiation in cancer cells may be the reason behind this positive outcome [68,69,70]. Although the survival analysis did not achieve significance, we found that an increased expression of the PIAS3 protein was related to a shorter survival. This could be confirmed by the results obtained by Yang et al., who described that an increased total PIAS3 protein level may predict a poor prognosis [71]. These findings indicate that PIAS3 can be considered as an oncogenic protein involved in tumorigenesis and may influence patient outcomes. A higher expression of SOCS3 significantly reduced the risk of metastasis. Many retrospective studies underlined that an elevated SOCS3 expression significantly correlated with DFS and OS in patients with BC, colorectal cancer, gastric cancer, ovarian cancer, and prostatic cancer [52]. Analysis using an ROC plotter suggested that BC patients with high expression levels of SOCS3 were less sensitive to chemotherapy [36]. We did not find a significant relationship between SOCS3 expression and BC patient survival in this study.

Considering the above facts, we can speculate that both proteins simultaneously participate in STAT5 functions, but only PIAS3 can be related to PIP. 

In the fluorescence images, we noted significantly higher levels of STAT5, STAT5-P, and PRLR in cells with silenced PIAS3 compared to the controls. This was the expected effect of the reduced level of PIAS3, known as the negative regulator of STATs and PRLR [16]. Incubation with PRL relocated STAT5 and STAT5-P to the cell nucleus, where they acted as transcriptional factors. Moreover, PRL treatment abolished the inhibitory effect of PIAS3 by increasing the fluorescent signal for PRLR and STAT5 in T47D CTRL cells. As McHale described, PRL in complex with the peptidyl-prolyl isomerase cyclophilin B acts as a transcriptional inducer, mediating the release of PIAS3 from the prolactin-induced latent transcription factor STAT5 [16]. 

According to the proposed hypothesis, an increased level of STAT5 should promote the upregulation of PIP transcription, thereby enhancing the sensitivity of cells to DOX. Unfortunately, there were no significant differences in the case of PIP levels, both at the mRNA and protein levels. Unexpectedly, the level of PIP was lower in cells with silenced PIAS3 inhibitors. We can assume that PIAS3 silencing, despite its positive effect on STAT5, does not positively affect the expression of PIP and requires additional co-stimulation, e.g., DHT acting on androgen receptors. Interestingly, some studies have shown that STAT5 activation inhibits PRL-induced activating protein-1 (AP-1) activity, which may play a role in PIP expression [7,72]. One mechanism for regulating PIP translation involves RUNX2. The *RUNX2* gene has a regulatory region that binds AP1 regulated by mitogen-activated protein kinase (MAPK) [73]. In the previous publication, we hypothesized that extracellular PIP, through its unknown receptor, induces the MAPK signaling cascade, amplifying pro-apoptotic signals activated in response to the genotoxic effect of cytostatics [14]. This may explain why we observed lower PIP levels in T47D shPIAS3 cells despite the expected increase in STAT5.

PRL plays a crucial role in promoting the survival and motility of breast epithelial cells within malignant tissues by increasing the proliferation of BC cells and actively inhibiting the apoptosis of mammary tumor cells [16]. This effect was visible in the MTT test, where incubation with PRL increased the cell viability in both T47D CTRL and T47D shPIAS3 cells. Howell et al. observed that DOX induced the expression of PRL mRNA and protein in BC cell lines. Additionally, PRL acts as a survival factor [74]. These observations clarify the increased expression of STAT5 at the mRNA and protein levels after treatment with DOX, and PRL with DOX. PRL, in a paracrine/autocrine manner, protects against the cytotoxic effect of chemotherapy. Interestingly, T47D CTRL cells, with a higher level of PIP, were more sensitive to DOX than T47D shPIAS3 cells [74]. This is another argument proving the lack of a positive influence of PIAS3 on PIP levels and cell sensitivity to chemotherapy. This result also confirms our previous observations that cells with a higher PIP expression (T47D CTRL) are more sensitive to the effects of DOX [7]. Multi-omics data published by Zhang show that the sensitivity of cells to chemotherapy varies depending on the expression of PIAS3 [75]. In BC cells, PIAS3 expression is negatively correlated with sensitivity to dacarbazine, while it is positively correlated with sensitivity to mitoxantrone. No significant correlation was found for DOX [75]. DOX induces the production of reactive oxygen species, which can cause further damage to cellular components and activate molecular signals that lead to apoptosis [76]. This is crucial, because our last microarray analysis revealed a significant increase in the expression of genes associated with anti-proliferative and pro-apoptotic effects in PIP-expressing BC cells. We suspect that an increased sensitivity to chemotherapy results from PIP’s pro-apoptotic activity, evidenced by the upregulation of specific pro-apoptotic genes [14]. Interestingly PIAS belongs to the cellular inhibitor of the apoptosis protein-1 (c-IAPs) class of proteins, which regulate the overall frequency of apoptosis during normal homeostasis, including cell survival and tissue turnover. c-IAPs and PIAS can negatively regulate apoptosis through several mechanisms like inhibiting caspase activity, neutralizing the activation of pro-caspases, and acting as ubiquitin ligases, promoting the proteasomal degradation of pro-apoptotic proteins [77]. 

In conclusion, the PIP, SOCS3, PIAS3, and STAT5 expression patterns in non-cancerous and BC tissues were analyzed by tissue microarray-based immunohistochemical staining and real-time PCR at the mRNA level. The analyzed STAT inhibitors showed heterogeneity in their expression. The results revealed that PIP and STAT5 significantly decreased as the tumor grades, size, and TNM increased. By contrast, the expression levels of nuclear PIAS3 tended to increase as the tumor progressed. Both STAT inhibitors seemed to be related to the status of ER and PR receptors. Statistical analyses indicated that the expression of nuclear PIAS3, rather than SOCS3, was correlated with PIP. STAT5 seemed to correlate positively with PIP, SOCS3, and PIAS3 and can be regarded as a favorable prognostic factor for the growth and survival of BC cells. The decreased expression of PIAS3 can be related to a better prognosis in BC patients. We suppose that PIAS3, rather than SOCS3, could have biological implications in BC. Nevertheless, in vitro studies did not confirm the positive effect of PIAS3 silencing on PIP expression and cell response to DOX. The regulation of PIP expression might be the effect of the complex interactions of PRL with other important hormones, cytokines, and growth factors in BC. The obtained results encourage further searches for factors influencing the regulation of PIP and its role in the response of BC cells to chemotherapy.

## 4. Materials and Methods

### 4.1. Patients’ Characteristics

Studies were conducted using archival tissue material from 554 cases of IDC. The material was collected in the years 2004–2022. It was obtained from the Institute of the Mother of Poland Health Center in Łódź, the Oncology Center of the Institute of Maria Skłodowskiej-Curie in Krakow, and the Breast Unit of Lower Silesian Oncology, Pulmonology and Hematology Center in Wroclaw. The control tissue included 61 mastopathy samples from the 4th Military Clinical Hospital in Wroclaw. A histopathological evaluation of the preparations stained with hematoxylin and eosin was used to determine the tumor type and assess the histological grade of G tumors according to the World Health Organization criteria of the 8th edition [78]. Each case also had clinical and pathological data such as T, N, and distant M metastases. The status of ER+, PR+, and HER2 receptors was also assessed. The clinicopathological characteristics of the patients are presented in Table 2. The patients were aged between 29 and 82 years (mean age: 57.8 years). The patients were followed up with an average time of 58.29 ± 19.08 months (range 1–169).

In addition, tissue samples, including 42 BC samples, 40 control tissues obtained from tumor margins, and 16 mastopathy samples, were preserved in RNA later (ThermoFisher Scientific, Waltham, MA, USA). The patients whose material was used gave written informed consent. The study was approved by the Wroclaw Medical University Institutional Review Board and the Bioethics Committee (No. KB-731/2019).

### 4.2. TMAs—Tissue Microarrays 

Twenty tissue microarrays (TMAs) were prepared from 554 paraffin blocks with BC tissue sections. Two TMAs were also prepared from mastopathy samples (control). For this purpose, histological preparations were made from archival samples stained with hematoxylin and eosin. The slides were scanned using a Panoramic Midi II histological scanner (3DHISTECH Ltd., Budapest, Hungary). Cancer sites with a core size of 1.5 mm were selected by the Panoramic Viewer (3DHISTECH Ltd.) and were transferred to the tissue arrays using the TMA Grand Master (3DHISTECH Ltd.).

### 4.3. Immunohistochemistry 

IHC reactions were carried out on TMA 4 µm thick paraffin sections using the automatic DAKO Autostainer Link48 (Dako/Agilent Technologies, Santa Clara, CA, USA). Deparaffinization, hydration, and thermal epitope demasking were performed using low (Ki-67, PIAS3)/high (PIP, SOCS3, STAT5, PRLR) pH Target Retrieval Solution (Dako/Agilent Technologies) for 20 min at 97 °C in a Dako PT Link (Dako/Agilent Technologies) apparatus. The EnVision™ Detection System, Peroxidase/DAB+, Rabbit/Mouse kit (Dako/Agilent Technologies) was used to visualize the antigen. To evaluate the immunohistochemical levels of the proteins, sections were incubated for 20 min at room temperature (RT) with the following primary antibodies: anti-GCDFP-15 mouse monoclonal antibody (clone 23A3, ready to use (RTU) code no. IR077, Dako/Agilent Technologies), anti-Ki-67 mouse monoclonal antibody (clone MIB-1, RTU, IR626, Dako/Agilent Technologies), anti-PIAS3 rabbit polyclonal antibody (1:500, code no. ab58406, Abcam, Cambridge, UK), anti-SOCS3 rabbit polyclonal antibody (1:450, code no. ab16030, Abcam), anti-STAT5 recombinant rabbit monoclonal antibody (1:6800, code no. ab194898, Abcam), anti-STAT5-P (phospho Y694) antibody [E208] recombinant rabbit monoclonal antibody (1:2000, code no. ab ab32364, Abcam), and recombinant rabbit monoclonal antibody anty-PRLR (clone EPR7184(2), 1:800, code no. ab 170935, Abcam). The sections were counterstained with FLEX Hematoxylin (Dako/Agilent Technologies). Negative control sections were used with the absence of the primary antibody. Two independent investigators analyzed the expression levels of the antigens at a magnification of ×200 using a BX41 (Olympus, Tokyo, Japan) light microscope coupled with a DP-12 camera and the CellD (Olympus) version 3.4 software for computer image analysis. Positive IHC reactions for GCDFP-15, PIAS3, SOCS3, and PRLR were assessed using Remmele and Stegner’s immunoreactive score (IRS) scale [79]. This scale (0–12 pts) evaluates the percentage of positive cancer cells (A) and the staining intensity of the reaction (B). The final result is the product of these two values (AxB). Additionally, the nuclear expression intensity for Ki-67, STAT5, and PIAS3 was determined using a scale that analyzes the percentage of the number of cancer cells with a positive nuclear expression of the antigens studied, according to the following scale: 0–5%—no reaction (0 p.), 5–10%—weak reaction (1 p.), 11–25%—moderate reaction (2 p.), 25–50%—medium reaction (3 p.), and over 50%—intense reaction (4 p.).

### 4.4. Real-Time PCR

The total RNA was isolated from IDC tumors and cell lines (control T47D CTRL and silenced T47D shPIAS3) using the RNeasy Mini kit (Qiagen, Hilden, Germany) according to the manufacturer’s instructions. The concentration and quality of the isolated RNA were measured on a NanoDrop1000 (Thermo-Fisher Scientific, Waltham, MA, USA). Reverse transcription reactions were performed using a high-capacity cDNA reverse transcription kit (Applied Biosystems, Foster City, CA, USA). Reactions were performed in triplicate and evaluated by real-time PCR using a 7500 Fast Real-Time PCR System, TaqMan System primers, and probes (Applied Biosystems). The primers and probes used in the reactions included the following: *PIP*: Hs00160082_m1 no. category 4351370, *PIAS3*, Hs00180666_m1, catalog no. 4351370; *SOCS3*, Hs02330328_s1, catalog no. 4351370; *STAT5A*, Hs00234181_m1, no. 4351370, *STAT5B*, Hs00273500_m1, catalog no. 4351370; and *ACTB* Hs99999903_m1 for β-actin (Applied Biosystems). The thermal cycling conditions were as follows: polymerase activation at 50 °C for 2 min, initial denaturation at 94 °C for 10 min, denaturation at 94 °C for 15 s, annealing of primers and probes, and synthesis at 60 °C for 1 min by 40 cycles. The results were normalized to the expression of the β-actin reference gene. The RQ of mRNA was calculated by the ΔΔCt method. The experiments were repeated three times.

### 4.5. Cell Line

The T47D cell line was purchased from the European Type Cell Culture Collection (Sigma-Aldrich/MilliporeSigma, Burlington, MA, USA). T47D cells were cultured in RPMI 1640 medium (ThermoFisher Scientific). The media were supplemented with 10% fetal bovine serum (FBS) (ThermoFisher Scientific), 2 mM l-glutamine, penicillin (100 μg/mL), streptomycin (100 U/mL) (Sigma-Aldrich/MilliporeSigma), and human insulin (0.2 U/mL) (Catalog No. 12585–014; Thermo Fisher Scientific).

LentiX 293T cells were purchased from Clontech Laboratories (Mountain View, CA, USA) and cultured in αMEM supplemented with 10% FBS (ThermoFisher Scientific), 2 mM L-glutamine, 100 U/mL of streptomycin, and 100 μg/mL of penicillin. The cell culture conditions were 37 °C and a 5% CO_2_ concentration.

Cells (T47D CTRL and T47D shPIAS3) for MTT, RT-PCR, and WB were maintained in phenol red-free and serum-free RPMI 48 h before stimulation with 1 ng/mL of PRL (human recombinant PRL expressed in *E. coli*) (Sigma-Aldrich/MilliporeSigma, catalog no. L4021; half maximal effective concentration (EC_50_): 0.030–0.300 ng/mL). The half maximal inhibitory concentration (IC_50_) values of DOX were calculated using the IC_50_ Tool Kit (T47D CTRL IC_50DOX_ = 0.47 µM, T47DshPIAS3 IC_50DOX_ = 0.38 µM). Cultures were then incubated in the absence or presence of PRL (20 min) and subsequently in the presence of increasing concentrations of DOX (Sigma-Aldrich/MilliporeSigma, catalog no. D1515) for 48 h.

### 4.6. Virus Production, Transductions, and Cell Maintenance

For lentivirus production packaging, LentiX 293T cells were co-transfected at 50–60% confluence with 20 μg of PIAS3 MISSION shRNA (SHCLNG-NM_006099, TRCN0000020786) expression vector or MISSION pLKO.1-PURO shRNA Control Plasmid DNA (SHC02–1EA) (MISSION^®^ shRNA Plasmid DNA system, Sigma-Aldrich/MilliporeSigma), packaging vector psPAX2 (15 µg), and VS.V-G protein vector pMD2.G (5 µg) (Addgene, Watertown, MA, USA) using polyethyleneimine (Sigma-Aldrich/MilliporeSigma) at a concentration of 1 mg/mL. Culture supernatants containing virus particles were collected 72 h after transfection and clarified through a 0.45 μm pore size filter (Sigma-Aldrich/MilliporeSigma). Virus-containing supernatants were concentrated 100X on an Amicon Ultra-15K:100,000 (Sigma-Aldrich/MilliporeSigma). T47D cells (2 × 10^4^) were transduced with the concentrated virus stock by centrifugation (2460× *g*) at 23 °C for 2.5 h. The cells were selected for puromycin (Thermo Fisher Scientific) resistance (1 µg/mL) for one week and maintained in a medium containing 1 µg/mL of puromycin.

### 4.7. Immunofluorescence 

For 24 h microculture, 600 µL of 2 × 10^4^ cells per well was set up Millicell EZ 8-well glass slides (Sigma-Aldrich/MilliporeSigma) and incubated at 37 °C. Cells were grown in the absence or presence of 1 ng/mL of PRL (Merc/Sigma) for 20 min. Subsequently, cells were fixed with 4% paraformaldehyde for 12 min at RT and permeabilized using 0.2% Triton X-100 for 10 min. After blocking with 3% bovine serum albumin in phosphate-buffered saline (PBS) with Tween 20 for 1 h, the cells were incubated overnight at 4 °C with the following primary antibodies: anti-GCDFP-15 recombinant rabbit monoclonal antibody (clone EP1582Y, 1:50, Abcam), anti-PIAS3 mouse monoclonal (1:50, code no. sc-46682, Santa Cruz Biotechnology, Dallas, TX, USA), anti-STAT5 recombinant rabbit monoclonal antibody (1:250, code no. ab194898, Abcam), anti-STAT5-P (phospho Y694) recombinant rabbit monoclonal antibody (clone E208, 1:250, code no. ab32364, Abcam), and recombinant rabbit monoclonal antibody anty-PRLR (clone EPR7184(2), 1:250, code no. ab170935, Abcam). Subsequently, the slides were incubated for 1 h at RT with donkey anti-rabbit secondary Alexa Fluor 568 conjugated antibody (1:2000; code no. ab175470; Abcam) or donkey anti-mouse secondary Alexa Fluor 488 conjugated antibody (1:2000; clone, code no. ab1501; Abcam). The sections were mounted in ProLong Diamond Antifade Reagent (Thermo Fisher Scientific) and analyzed using a confocal laser scanning microscope FV3000 Fluoview (Olympus). Omitting the addition of primary antibodies was performed to obtain the respective negative controls. The fluorescence intensity was quantified by measuring the brightness of the emitted light in 10 hot spots using Fluoview version 2.5.1 software, which calculates the fluorescence intensity for each region of interest. The result was the average of the measurements.

### 4.8. Western Blot

The Western blot method measured the protein expression levels in the cytoplasmic and nuclear fractions in the control and silenced T47D cell lines. In total, 5–6 × 10^6^ cells in the exponential growth phase were taken for each analysis. After washing with ice-cold PBS, cell fractions were isolated according to the manufacturer’s instructions using the Subcellular Protein Fractionation Kit for Cultured Cells (Thermo Fisher Scientific, code no. 78840). The concentration of isolated cell lysate protein was measured using the Pierce BCA Protein Assay Kit (Thermo Fisher Scientific) and a NanoDrop 1000 spectrophotometer (Thermo Fisher Scientific). Proteins were denatured at 95 °C for 10 min in sample loading buffer GLB (250 mM Tris-HCl, 40% glycerol, 20% β-mercaptoethanol, 8% SDS and bromophenol blue), transferred to PSQ membrane (Sigma-Aldrich/MilliporeSigma), and blocked with 5% non-fat milk (Bio-Rad, Marnes-la-Coquette, France) in Tris-buffered saline (TBS) for 1 h at RT. The membrane was incubated with the following primary antibodies overnight at 4 °C: anti-GCDFP-15 recombinant rabbit monoclonal antibody (clone EP1582Y, 1:1000, Abcam), anti-PIAS3 mouse monoclonal antibody(1:500, code no. sc-46682, Santa Cruz Biotechnology), anti-STAT5 recombinant rabbit monoclonal antibody (1:1000, code no. ab194898, Abcam), and anti-STAT5-P (phospho Y694) recombinant rabbit monoclonal antibody (clone E208, 1:1000, code no. ab32364, Abcam). Subsequently, the membrane was incubated with the secondary horseradish peroxidase conjugated with donkey anti-rabbit antibody diluted in 0.5% milk in 0.1% TBS with 0.1% Tween 20 (1:3000; code no. 711–035-052; Jackson ImmunoResearch, Cambridgeshire, UK) for 1h at RT. The proteins were visualized using the Luminata Classico Western HRP Substrate (Sigma-Aldrich/MilliporeSigma). The membrane was stripped and incubated with monoclonal mouse anti-actin antibody (1:500, clone AC-40, code no. A4700, Merck, Darmstadt, Germany) used as the loading control. The data were documented for exposure times ranging from 2 s to 30 min in the Chemi-Doc XRS Molecular Imager apparatus (Bio-Rad). 

### 4.9. Analysis of Cell Viability by the MTT (3-(4,5-Dimethylthiazol-2-yl)-2,5-Diphenyltetrazolium Bromide)

Native and silenced T47D cells (5 × 10^3^) were grown in 96-well plates (Greiner BioOne, Kremsmünster, Austria). The cells were grown in the absence or presence of 1 ng/mL of PRL (Merck) for 20 min and in the presence of increasing concentrations of DOX (Merck) for 48 h. After the indicated periods, thiazolyl blue tetrazolium bromide solution (MTT, 5 mg/mL) (Merck) was added to each well, and the cells were cultured for an additional 4 h. After this time, the medium was removed, and the formed MTT-formazan crystals were dissolved in 0.1 mL of dimethyl sulfoxide for 30 min. The absorbance was measured with a Tecan microplate reader (Tecan Trading AG, Männedorf, Switzerland) at a wavelength of 550 nm. The experiments were repeated three times.

### 4.10. Statistical Analysis

The Kolmogorov–Smirnov test was used to evaluate the normality assumptions of the examined groups. The Mann–Whitney (tumor vs. control, ER+ vs. ER−, PR+ vs. PR−, HER+ vs. HER−; pN+ vs. pN−), Kruskal–Wallis test, two-way ANOVA with Dunn’s or Bonferroni multiple comparison tests (G, pT, TNM, molecular types; MTT test), and MANOVA with Bonferroni post hoc tests (to measure the effect of treatment on mRNA (RQ) levels in T47D cell lines) were used to statistically analyze the differences among all groups of patients and the clinicopathological data. Spearman’s rank correlation was used to test the correlations. The Kaplan–Meier method was used to construct survival curves. The Gehan–Breslow–Wilcoxon method and the univariate and multivariate Cox analyses of survival were performed to evaluate the analysis of survival. All the results were considered to be statistically significant when *p* < 0.05. Statistical analyses were conducted using Prism 5.0 (GraphPad, La Jolla, CA, USA), Statistica 13.1 (StatSoft, Cracow, Poland), and Origin(Pro), Version 2024b (OriginLab Corporation, Northampton, MA, USA).

## Figures and Tables

**Table 1:** Univariate and multivariate Cox proportional hazards analysis in 554 patients with invasive ductal breast carcinoma, (**A**) overall survival and (**B**) disease-free survival.

**(A)**	**Overall Survival (OS)**					
**Clinicopathological Parameters**	**Univariate**			**Multivariate**		
	***p*** **-Value**	**HR**	**95% HR CI**	***p*** **-Value**	**HR**	**95% HR CI**
Age (<60 vs. ≥60)	0.069	1.229	0.196–7.707	-	-	-
Tumor size (pT1 vs. pT2–T4)	0.239	1.789	0.679–4.714	-	-	-
Lymph nodes (pN− vs. pN+)	0.999	0.99	0.196–5.108	-	-	-
Clinical stage TNM (I–II vs. III–IV)	**0.042**	7.726	0.13–62.345	0.981	1.005	0.641–1.575
Grade of malignancy (G1, G2 vs. G3)	0.563	1.774	0.392–1.829	-	-	-
Estrogen receptor (ER− vs. ER+)	0.448	1.063	0.511–2.212	-	-	-
Progesterone receptor (PR− vs. PR+)	0.338	1.767	0.922–3.387	-	-	-
HER2 (negative vs. positive)	0.110	3.025	0.778–11.766	-	-	-
Ki-67 (<25% vs. ≥25%)	**0.037**	8.426	1.127–62.976	0.889	1.052	0.516–2.145
PIP (‘low’ vs. ‘high’)	0.29	0.523	0.154–1.777	-	-	-
PIAS3 cytoplasmic (‘low’ vs. ‘high’)	0.98	1.008	0.296–3.866	-	-	-
PIAS3 nuclear (PIAS3− vs. PIAS3+)	0.08	3.414	0.856- 13.603	-	-	-
SOCS3 (SOCS3− vs. SOCS3+)	0.812	0.812	0.147–4.482	-	-	-
STAT5 cytoplasmic (‘low’ vs. ‘high’)	**0.032**	0.502	0.107–2.347	0.171	0.686	0.400–1.177
STAT5 nuclear(STAT5 nuclear− vs. STAT5 nuclear+)	**0.011**	0.355	0.065–1.947	0.27	1.409	0.759–2.615
STAT5-P (‘low’ vs. ‘high’)	0.859	1.091	0.416–2.859	-	-	-
PRLR (‘low’ vs. ‘high’)	0.524	1.464	0.452—4.748	-	-	-
**(B)**	**Disease-Free Survival (DFS)**					
**Clinicopathological Parameters**	**Univariate**			**Multivariate**		
	***p*** **-Value**	**HR**	**95% HR CI**	***p*** **-Value**	**HR**	**95% HR CI**
Age (<60 vs. ≥60)	0.107	1.191	0.192–7.403	-	-	-
Tumor size (pT1 vs. pT2–T4)	0.271	1.679	0.666–4.233	-	-	-
Lymph nodes (pN− vs. pN+)	0.966	1.03	0.216–4.946	-	-	-
Clinical stage TNM (I–II vs. III–IV)	0.055	1.726	0.957–2.345	-	-	-
Grade of malignancy (G1, G2 vs. G3)	0.272	1.823	0.396–2.710	-	-	-
Estrogen receptor (ER− vs. ER+)	0.121	1.605	0.133–2.777	-	-	-
Progesterone receptor (PR− vs. PR+)	0.529	1.546	0.083–3.584	-	-	-
HER2 (negative vs. positive)	**0.047**	3.669	1.017–13.233	**0.011**	2.008	1.176–3.429
Ki-67 (<25% vs. ≥25%)	**0.034**	8.108	1.172–56.102	0.801	0.943	0.600–1.483
PIP (‘low’ vs. ‘high’)	0.25	0.503	0.153–1.656	-	-	-
PIAS3 cytoplasmic (‘low’ vs. ‘high’)	0.81	0.868	0.263–2.866	-	-	-
PIAS3 nuclear (PIAS3− vs. PIAS3+)	0.13	2.764	0.736–10.377	-	-	-
SOCS3 (SOCS3− vs. SOCS3+)	0.895	0.898	0.185–4.376	-	-	-
STAT5 cytoplasmic (‘low’ vs. ‘high’)	**0.041**	0.539	0.105–2.752	0.157	0.684	0.405–1.157
STAT5 nuclear(STAT5 nuclear− vs. STAT5 nuclear+)	**0.002**	0.253	0.041–1.564	0.360	1.355	0.707–2.599
STAT5-P (‘low’ vs. ‘high’)	0.954	0.951	0.318–2.843	-	-	-
PRLR (‘low’ vs. ‘high’)	0.696	1.601	0.746–3.436	-	-	-

Significant *p*-values are given in bold. CI, confidence interval; ER, estrogen receptor; G, grade of malignancy; HER2, human epidermal growth factor receptor; HR, hazard ratio; Ki-67, cellular marker for proliferation; PIAS3, protein inhibitor of activated STAT3; PIP, prolactin-induced protein; PR, progesterone receptor; PRLR, prolactin receptor; SOCS3, suppressor of cytokine signaling 3; STAT5, signal transducer and activator of transcription 5; STAT5-P, phosphorylated signal transducer and activator of transcription 5; TNM, tumor/node/metastasis clinical stage; pN, lymph nodes status; pT, tumor size.

**Table 2:** Clinicopathological characteristics of BC.

Clinicopathological Parameters	IHC Patients (*n* = 554)	RT-PCR Patients (*n* = 42)
**Age**		
≤50	99 (18%)	9 (19%)
>50	455 (82%)	33 (81%)
**Tumor size**		
pT1	255 (46%)	23 (55%)
pT2	272 (49%)	16 (38%)
pT3–T4	27 (5%)	3 (7%)
**Lymph nodes (N)**		
pN0	316 (57%)	25 (60%)
pN1–N3	227 (41%)	13 (32%)
pNx	11 (2%)	4 (8%)
**TNM clinical stage**		
I	161 (29%)	15 (35%)
II	238 (43%)	18 (44%)
III	67 (12%)	6 (15%)
IV	88 (16%)	3 (6%)
**Grade of malignancy**		
G1	89 (16%)	7 (17%)
G2	299 (54%)	24 (57%)
G3	166 (30%)	11 (26%)
**Estrogen receptor**		
Negative	144 (26%)	10 (23%)
Positive	410 (74%)	32 (77%)
**Progesterone receptor**		
Negative	194 (35%)	15 (35%)
Positive	360 (65%)	27 (65%)
**HER2**		
Negative	343 (62%)	28 (67%)
Positive	211 (38%)	14 (33%)
**Ki-67**		
≤25	327 (59%)	27 (64%)
>25	227 (41%)	15 (36%)
**Molecular tumor types**		
Triple-negative	55 (10%)	9 (14%)
Other types	499 (90%)	33 (86%)
**Hormonal therapy**		
Negative	95 (17%)	7 (17%)
Positive	262 (47%)	35 (83%)
No data	197 (36%)	
**Chemotherapy**		
Negative	197 (36%)	20 (48%)
Positive	161 (29%)	22 (52%)
No data	194 (35%)	

G, grade of malignancy; HER2, human epidermal growth factor receptor; IHC, immunohistochemistry, Ki-67, cellular marker for proliferation; RT-PCR, real-time PCR; TNM, tumor/nodes/metastasis clinical stage; pN, lymph nodes status; pT, tumor size.

## Data Availability

The raw data and the analytic methods will be made available to other researchers for purposes of reproducing the results in their own laboratories upon reasonable request. To access protocols or datasets contact karolina.jablonska@umw.edu.pl.

## Supplementary Materials

The following supporting information can be downloaded at: https://www.mdpi.com/article/10.3390/ijms26041416/s1.
