# Supplementary material for: Association of Selected STAT Inhibitors with Prolactin-Induced Protein (PIP) in Breast Cancer
Source: Int J Mol Sci. 2025 Feb 7;26(4):1416. doi: 10.3390/ijms26041416 (PMC11855718; doi:10.3390/ijms26041416)
Supplement: Supplementary file 1 [file ijms-26-01416-s001.zip › ijms-3383764-supplementary.pdf]

*Article*

# Association of Selected STAT Inhibitors with Prolactin-Induced Protein (PIP) in Breast Cancer

**Karolina Jabłońska [1], Alicja Kmiecik [1], Katarzyna Nowińska [1], Aleksandra Piotrowska [1], Jarosław Suchański [2],Katarzyna Ratajczak-Wielgomas [1], Aleksandra Partyńska [1], Hanna Romanowicz [3], Beata Smolarz [3], Rafał Matkowski [4,5] and Piotr Dzięgiel [1,6]**

1. Division of Histology and Embryology, Department of Human Morphology and Embryology, Faculty of Medicine, Wroclaw Medical University, 50–368 Wroclaw, Poland; alicja.kmiecik@umw.edu.pl (A.K.), katarzyna.nowinska@umw.edu.pl (K.N.); aleksandra.piotrowska@umw.edu.pl (Aleksandra Piotrowska); katarzyna.ratajczak-wielgomas@umw.edu.pl (K.R-W.), aleksandra.partynska@umw.edu.pl (Aleksandra Partyńska); piotr.dziegiel@umw.edu.pl (P.D.)
2. Department of Biochemistry and Molecular Biology, Wroclaw University of Environmental and Life Sciences, 50–375 Wroclaw, Poland; jaroslaw.suchanski@upwr.edu.pl (J.S.)
3. Department of Pathology, Polish Mother Memorial Hospital-Research Institute, 93–338 Lodz, Poland; hanna-romanowicz@wp.pl (H.R.); smolbea@wp.pl (B.S.)
4. Department of Oncology, Faculty of Medicine, Wroclaw Medical University, 50–367 Wroclaw, Poland; rafal.matkowski@umw.edu.pl (R.M.)
5. Lower Silesian Oncology, Pulmonology and Hematology Center, 53–413, Wroclaw, Poland
6. Department of Human Biology, Faculty of Physiotherapy, Wroclaw University of Health and Sport Sciences, 51–612 Wroclaw, Poland

* Correspondence: karolina.jablonska@umw.edu.pl (K.J.); Tel.: +48–71–784–16-80;

**Table S1.** Median overall survival (mOS) and disease-free survival (mDFS) for clinicopathological factors.

| Clinicopathological parameter | **Median OS (months)** | **Median DFS (months)** |
|---|---|---|
| Age (<60 ) | not reached | not reached |
| Age (⩾60) | not reached | not reached |
| Tumor size (pT1 ) | not reached | not reached |
| Tumor size (pT2-T4) | not reached | not reached |
| Lymph nodes (pN-) | 137 | 104 |
| Lymph nodes (pN+) | 96 | 131 |
| Clinical stage TNM (I-II) | not reached | not reached |
| Clinical stage TNM (III-IV) | 96 | 79 |
| Grade of malignance (G1,G2) | 137 | 104 |
| Grade of malignance (G3) | 96 | 131 |
| Estrogen receptor (ER-) | 108 | 104 |
| Estrogen receptor (ER+) | 96 | 172 |
| Progesterone receptor (PR-) | 94 | not reached |
| Progesterone receptor (PR+) | 137 | 104 |
| HER2 (negative) | 137 | 104 |
| HER2 (positive) | 108 | not reached |
| Ki67 (<25%) | not reached | |
| Ki67 ( ⩾25%) | 96 | 79 |
| PIP (‘low’) | 108 | 131 |
| PIP (‘high’) | 94 | not reached |
| PIAS3 cytoplasmic (‘low’) | not reached | not reached |

| | | |
|---|---|---|
| PIAS3 cytoplasmic ('high') | not reached | not reached |
| PIAS3 nuclear (PIAS3-) | not reached | not reached |
| PIAS3 nuclear (PIAS3+) | not reached | not reached |
| SOCS3 (SOCS3-) | not reached | not reached |
| SOCS3 (SOCS3+) | not reached | not reached |
| STAT5 cytoplasmic ('low') | not reached | not reached |
| STAT5 cytoplasmic ('high') | not reached | not reached |
| STAT5 nuclear (STAT5 nuclear-) | not reached | not reached |
| STAT5 nuclear (STAT5 nuclear+) | not reached | not reached |
| STAT5 P ('low') | not reached | not reached |
| STAT5 P ('high') | not reached | not reached |
| PRLR ('low') | 96 | 79 |
| PRLR ('high') | 108 | 131 |

ER, estrogen receptor; G, grade of malignance; HER2, human epidermal growth factor receptor; Ki-67, cellular marker for prolif-eration; PIAS3, protein inhibitor of activated STAT3; PIP, prolactin-induced protein; PR, progesterone receptor; PRLR, prolactin receptor; SOCS3, suppressor of cytokine signaling 3; STAT5, signal transducer and activator of transcription 5; STAT5-P, phosphorylated signal transducer and activator of transcription 5; TNM, tumor/nodes/metastasis clinical stage; pN, lymph nodes status; pT, tumor size.

**Table S2.** Analysis of the MANOVA model significance to measure the effects of PRL, DOX, and PRL+DOX on the mRNA levels of the tested markers in T47D shPIAS3 and T47D CTRL cells.

| Test | Test Value | F Value | NumDF | DenDF | Prob>F | Sig |
|---|---|---|---|---|---|---|
| Wilks' Lambda | 0.088 | 4.355 | 12 | 34 | 3.477E-4 | 1 |
| Hotelling-Lawley Trace | 7.507 | 7.298 | 12 | 35 | 1.846E-6 | 1 |
| Pillai's Trace | 1.164 | 2.379 | 12 | 45 | 0.018 | 1 |
| Roy's Largest Root | 7.104 | 26.641 | 4 | 15 | 1.159E-6 | 1 |
